# Sphingosine kinase 1 is involved in triglyceride breakdown by maintaining lysosomal integrity in brown adipocytes

**DOI:** 10.1016/j.jlr.2023.100450

**Published:** 2023-09-24

**Authors:** Jun-ichi Morishige, Kazuaki Yoshioka, Hiroki Nakata, Kazuhiro Ishimaru, Naoto Nagata, Tamotsu Tanaka, Yoh Takuwa, Hitoshi Ando

**Affiliations:** 1 Department of Cellular and Molecular Function Analysis, Graduate School of Medical Sciences, Kanazawa University, Kanazawa, Japan; 2 Department of Physiology, Graduate School of Medical Sciences, Kanazawa University, Kanazawa, Japan; 3 Department of Clinical Engineering, Faculty of Health Sciences, Komatsu University, Komatsu, Japan; 4 Graduate School of Technology, Industrial and Social Sciences, Tokushima University, Tokushima, Japan

**Keywords:** Adipose tissue, brown, lipid droplets, lipase, lipolysis, lysosome, sphingolipids, sphingosine kinase, sphingosine phosphate, thermogenesis, triglyceride

## Abstract

Sphingosine 1-phosphate (S1P) has been implicated in brown adipose tissue (BAT) formation and energy consumption; however, the mechanistic role of sphingolipids, including S1P, in BAT remains unclear. Here, we showed that, in mice, BAT activation by cold exposure upregulated mRNA and protein expression of the S1P-synthesizing enzyme sphingosine kinase 1 (SphK1) and S1P production in BAT. Treatment of wild-type brown adipocytes with exogenous S1P or S1P receptor subtype-selective agonists stimulated triglyceride (TG) breakdown only marginally, compared with noradrenaline. However, genetic deletion of *Sphk1* resulted in hypothermia and diminished body weight loss upon cold exposure, suggesting that SphK1 is involved in thermogenesis through mechanisms different from receptor-mediated, extracellular action of S1P. In BAT of wild-type mice, SphK1 was localized largely in the lysosomes of brown adipocytes. In the brown adipocytes of *Sphk1*^−/−^ mice, the number of lysosomes was reduced and lysosomal function, including proteolytic activity, acid esterase activity, and motility, was impaired. Concordantly, nuclear translocation of transcription factor EB, a master transcriptional regulator of lysosome biogenesis, was reduced, leading to decreased mRNA expression of the lysosome-related genes in *Sphk1*^−/−^ BAT. Moreover, BAT of *Sphk1*^−/−^ mice showed greater TG accumulation with dominant larger lipid droplets in brown adipocytes. Inhibition of lysosomes with chloroquine resulted in a less extent of triglyceride accumulation in *Sphk1*^−/−^ brown adipocytes compared with wild-type brown adipocytes, suggesting a reduced lysosome-mediated TG breakdown in *Sphk1*^−/−^ mice. Our results indicate a novel role of SphK1 in lysosomal integrity, which is required for TG breakdown and thermogenesis in BAT.

Brown adipose tissue (BAT) differs from white adipose tissue (WAT) in its ability to generate energy via nonshivering thermogenesis. The thermogenic activity of BAT is mainly controlled by the sympathetic nervous system via β-adrenergic receptor signaling (1). BAT activation leads to lipid droplet breakdown and subsequent production of free fatty acids that are used as fuel for thermogenesis via mitochondrial uncoupling protein 1 (UCP1) (1). BAT activity decreases with aging, obesity, and type 2 diabetes (2) and inverse correlations have been shown between BAT activity and body mass index and visceral adiposity parameters, such as visceral fat area and waist circumference (3). Increased BAT activity due to cold exposure or pharmacological stimulation improves diet-induced obesity, dyslipidemia, glucose homeostasis, and insulin sensitivity (4, 5). Thus, maintaining active BAT and continuous heat production in BAT is an important therapeutic strategy to improve obesity.

Sphingosine 1-phosphate (S1P) is a bioactive sphingolipid produced via the action of two sphingosine kinase (SphK) isoenzymes, SphK1 and SphK2. Although both SphK1 and SphK2 synthesize the same product, they display different catalytic properties, subcellular localization, and tissue distribution (6). The subcellular localization of SphK1 and SphK2 is important to understanding the biological action of S1P. For instance, SphK1 translocates from the cytosol to the plasma membrane in response to hormones, cytokines, and S1P by itself. Intracellularly produced S1P is released into the extracellular fluid and acts as an extracellular mediator through five S1P-specific receptors (S1PR_1–5_) to induce various cellular responses, such as cell proliferation, cell motility, differentiation, and adhesion (7). S1P also acts intracellularly as functional molecules. It is produced by SphK1 in endosomes/autophagosomes and controls membrane trafficking and autophagy (8, 9, 10, 11, 12, 13). On the other hand, in the nucleus and mitochondria, S1P is generated by SphK2, where it is involved in DNA synthesis (14) and mitochondrial respiration (15), respectively.

Studies into the role of S1P in brown adipocytes are limited to date but are steadily increasing in number. For example, S1PR_2_ and S1PR_3_ are highly expressed in brown adipogenic progenitor cells and S1P regulates brown adipogenesis via S1PR_2_ (16). In addition, S1P regulates endothelial barrier function in BAT via S1PR_1_, controlling triglyceride (TG) uptake from blood into BAT. *Apom*^−/−^ mice, which show a 46% reduction in plasma S1P (17), display increased vascular permeability, increased TG uptake, increased energy consumption, and body weight reduction (17). These results suggest that extracellular S1P is involved in BAT formation and energy consumption in BAT. However, it is unclear how S1P influences BAT formation and energy expenditure in brown adipocytes.

The present study revealed that cold exposure of mice induces an approximately 2-fold rise in SphK1 expression, with an increased S1P content in BAT. We showed that SphK1 was mainly localized in lysosomes in adipocytes of BAT, and genetic deletion of *Sphk1* impaired lysosomal biogenesis and function, TG breakdown, and cold tolerance in BAT. Our findings suggest that SphK1 plays an important role in lipid droplet catabolism and energy consumption by controlling lysosomal biogenesis and function.

## Materials and Methods

### Animals

All animal experiments were performed using 8–10-week-old male *Sphk1*^+/+^ and *Sphk1*^−/−^ mice (18). *Sphk1*^−/−^ mice were kindly gifted by Dr Richard L. Proia at National Institute of Diabetes and Digestive and Kidney Diseases. *Sphk1*^−/−^ mice were backcrossed into the C57BL6/6J background more than 10 times. Mice were fed standard chow and water ad libitum and maintained in a 12 h/12 h light/dark cycle from 08:45–20:45 at 23°C. All animal experiments were performed according to procedures approved by the University Committee of Kanazawa University.

### Cold exposure experiment

Mice were individually housed at 4°C with access to food and water ad libitum. Cold exposure was started at the onset of light period. Body temperature was measured using a weighing environment logger equipped with a temperature probe for mice (A&D, Tokyo, Japan). A temperature probe was inserted 1 cm into the rectum, the temperature allowed to stabilize, and values were recorded. Mice were then anesthetized using pentobarbital and whole blood was collected by cardiac puncture. Tissues were dissected and immediately frozen in liquid nitrogen. Whole blood was drawn, kept for 1 h at room temperature, and then centrifuged at 4,600 *g* for 10 min at 4°C. The serum was then carefully collected. Plasma was prepared from whole blood drawn into a syringe treated with EDTA and then mixed with citrate-theophylline-adenosine-dipyridamole and EDTA to suppress the undesirable lysophospholipids formation (19). The tubes were immediately placed on ice and then centrifuged at 1,150 *g* for 5 min at 4°C and the plasma was carefully collected. Serum and plasma samples were stored at −80°C until analyzed.

### Blood chemistry

Serum TG, total cholesterol, and NEFA concentrations were measured using a LabAssay Triglyceride kit (Cat# 290-63701, FUJIFILM Wako Pure Chemical, Osaka, Japan), LabAssay Cholesterol kit (Cat# 294-65801, FUJIFILM Wako Pure Chemical), and LabAssay NEFA kit (Cat# 294-63601, FUJIFILM Wako Pure Chemical), respectively.

### Quantitative real-time PCR

Frozen BAT depots (around 15 mg) were homogenized in 1 ml of TRI Reagent (Cat# TR118, Molecular Research Center, OH) for 2 min using TissueLyser LT (Qiagen, Hilden, Germany) and zirconia beads. The homogenates were centrifuged at 12,000 *g* for 10 min at 4°C and the supernatants were transferred to 1.5-ml microcentrifuge tubes. Then, 0.2 ml of chloroform was added, vigorously vortexed, and centrifuged at 12,000 *g* for 10 min at 4°C. The upper layer was collected and total RNA was isolated using a FastGene RNA basic kit (Cat# FG-80050, NIPPON Genetics, Tokyo, Japan) according to the manufacture’s protocol. Purified RNA was quantified using a NanoDrop spectrophotometer (Thermo Fisher Scientific, MA) and cDNA was synthesized from 400 ng of RNA using High Capacity cDNA Reverse Transcription Kits (Cat# 4368814, Thermo Fisher Scientific). Quantitative real-time PCR analysis was performed using the ViiA7 real-time PCR system (Applied Biosystems) using Power SYBR Green Master Mix (Cat# A25777, Applied Biosystems, Thermo Fisher Scientific). The primer sequences used are listed in supplemental Table S1. The relative mRNA transcription level for each gene was calculated using the 2^−ΔΔct^ method normalized to the transcription of *Ppia*.

### Mitochondrial DNA determination

We determined mitochondrial DNA as described previously with minor modifications (20). In brief, total DNA was isolated from BAT using FastGene Genomic DNA Extraction Kit (Cat# FG-GD050T, NIPPON Genetics, Tokyo, Japan) according to the manufacture’s protocol. Purified DNA was quantified using a NanoDrop spectrophotometer (Thermo Fisher Scientific). Quantitative real-time PCR analysis was performed by the QuantStudio 6 Pro (Applied Biosystems, Thermo Fisher Scientific) using Power SYBR Green Master Mix. Relative mitochondrial DNA (mtDNA) content was expressed as ratio of the copy numbers of mtDNA-encoded gene (*mt-Co1*) to that of the nuclear DNA-encoded gene (*Ndufv1*) using the 2^−ΔΔct^ method. The primer sequences used are listed in supplemental Table S1.

### Western blotting

Frozen BAT was homogenized in 0.5 ml of RIPA buffer consisting of 10 mM Tris-HCl (pH 8.0), 140 mM NaCl, 0.1% sodium deoxycholate, 1 mM EDTA, 1% Triton X-100, 0.1% SDS, and 1 mM PMSF, protease inhibitor cocktail (Cat# 11836153001, Roche, Mannheim, Germany), and phosphatase inhibitor cocktail (1 mM Na_3_VO_4_, 10 mM β-glycerophosphate, and 10 mM NaF). Tissue homogenates were centrifuged at 15,000 *g* for 15 min at 4°C and supernatants were carefully collected avoiding the fat cake. Protein concentrations were determined using the BCA protein assay kit (Cat# 23225, Thermo Fisher Scientific). BAT lysates were separated by SDS-PAGE and transferred to PVDF membranes (Merck Millipore) using the Trans-Blot Turbo blotting system (Bio-Rad, CA). PVDF membranes were blocked with PVDF blocking reagent (Cat# NYPBR01, Toyobo, Osaka, Japan) for 1 h and then incubated with primary antibodies overnight at 4°C. Detailed information of the antibodies is described below (see “Antibodies for immunoblotting”). After washing with 0.1% Tween80 in Tris-buffered saline, the PVDF membranes were incubated with the appropriate alkaline phosphatase-conjugated secondary antibodies (1:1,000, anti-rabbit IgG: Cell Signaling Technology Cat# 7054, RRID:AB_2099235; 1:1,000, anti-mouse IgG: Cell Signaling Technology Cat# 7056, RRID, AB_330921; and 1:1,000, anti-rat IgG: Abcam Cat# Ab6846, RRID:AB_954684) for 1 h. Protein bands were visualized by the color reaction using the nitro blue tetrazolium (FUJIFILM Wako Pure Chemical) and 5-bromo-4-chloro-3′-indolylphosphate *p*-toluidine (FUJIFILM Wako Pure Chemical) system. Western blot band intensities were determined using Image Studio Lite software (LI-COR, version 5.2.5, RRID:SCR_013715) and quantified values were normalized using the value of β-actin or GAPDH as a loading control and expressed as multiples over the normalized values of controls.

### Immunofluorescence staining

Mice were intracardially perfused with PBS and 4% paraformaldehyde (PFA). BAT was then dissected from the interscapular region and fixed with 4% PFA in PBS overnight at 4°C. BAT was treated with PBS, 10% sucrose, and 20% sucrose in a stepwise manner and eventually incubated in Tissue-Tek OCT compound (Cat# 4583, Sakura Finetek Japan) containing 20% sucrose overnight at 4°C. After embedding in OCT compound containing 20% sucrose, the tissue block was cut into 15-μm-thick sections and mounted. BAT sections were dried at room temperature and permeabilized with acetone for 5 min. Lipid droplets were stained with BODIPY 493/503 (Cat# D3922, Thermo Fisher Scientific) as follows. BAT sections were permeabilized with 0.4% Triton X-100 in PBS for 15 min. BAT sections were washed three times with PBS and blocked with serum-free protein block (Cat# X0909, Dako, Agilent) for 30 min followed by incubation with primary antibodies overnight at 4°C. Details of the antibodies used are described below (see “Antibodies for immunostaining”). BAT sections were washed three times with 0.1% Tritone X-100 in PBS and incubated with Alexa fluorophore-conjugated secondary antibodies for 3 h at room temperature. For lipid droplet staining, BODIPY 493/503 was mixed with secondary antibodies. After washing three times with 0.1% Triton X-100 in PBS, BAT sections were incubated with DAPI (Cat# D1306, Molecular probes) in PBS for 30 min for cell nuclei staining, followed by washing twice with PBS. Thereafter, BAT sections were mounted with Fluoromount (Cat# K024, Diagnostic Biosystems, CA). Confocal microscopy was performed using an inverted Nikon Eclipse Ti2 confocal microscope (Nikon Instruments, Tokyo, Japan) attached to an Andor Dragonfly spinning-disk unit, Andor EMCCD camera (iXon DU888) (Andor Technologies, Belfast, Northern Ireland), and laser unit (Coherent). An oil-immersion objective (PlanApo360, numerical aperture 1.4; Nikon) was used for all experiments. Excitation for DAPI, Alexa Fluor 488, Alexa Fluor 568, and Alexa Fluor 647 chromophores was achieved using 405, 488, 561, and 637-nm lasers, respectively.

### S1P determination by liquid chromatography electrospray ionization tandem mass spectrometry

Frozen BAT depots (around 20 mg) were homogenized in 0.95 ml of chloroform/methanol/water (1:2:0.8, v/v) containing 50 pmol of C17-base S1P (Cat# 860651P, Avanti Polar Lipids, AL) by BioMasher II (Nippi). After centrifuging at 1,300 *g* for 5 min at 4°C, the supernatant was collected into a new glass tube and lipids were re-extracted from the remaining pellet using 0.95 ml of the same solvent. The remaining pellet was used for protein determination using the BCA protein assay kit. The collected supernatants were combined and mixed with 0.5 ml of chloroform, 0.5 ml of 5% KCl, and 0.005 ml of 28% aqueous ammonia for the formation of the two-phase system of the Bligh and Dyer method (21) under mild alkaline conditions. After vigorous shaking, the mixture was centrifuged at 1,300 *g* for 5 min at 4°C and the chloroform-rich layer was discarded. The upper phase was washed with chloroform/methanol (17:3, v/v) and 1 ml of the chloroform/methanol (17:3, v/v) and 0.02 ml of 5 M HCl were added. The mixture was vigorously shaken then centrifuged at 1,300 *g* for 5 min at room temperature. The lower chloroform phase was removed and the water/methanol phase was mixed with 0.5 ml of chloroform/methanol (17:3, v/v) for further extraction. The chloroform phases were combined and dried under a stream of nitrogen. Lipid extracts were redissolved in methanol, filtered, and dried. The lipid extracts were reconstituted with 0.1 ml of mobile phase with 25 nmol EDTA and applied for analysis using liquid chromatography electrospray ionization tandem mass spectrometry with a quadrupole-linear iontrap hybrid mass spectrometer, 4000Q TRAP (Applied Biosystem MDS Sciex, Concord, Ontario, Canada) and Agilent 1100LC system combined HTS-PAL autosampler (CTC Analytics AG, Zwingen, Switzerland) as described previously (22). Q1 and Q3 were set for the deprotonated molecular ion and [PO_3_]^-^ at *m/z* 79 for C18-base S1P. Concentrations of C18 S1P were calculated from the ratio of their peak negative ion areas to that of an internal standard.

Plasma (0.2 ml) was diluted with 0.25 ml of 5% KCl and mixed with 1 ml of chloroform/methanol (1:1, v/v) containing 50 pmol of C17-base S1P and 0.005 ml of 28% aqueous ammonia to form the two-phase system of Bligh and Dyer. S1P was extracted and quantified using same procedure as for BAT S1P.

### SphK activity assay

SphK activity was measured as described previously with minor modifications (23). Briefly, fresh BAT, inguinal WAT, and epidydimal WAT from 23°C-housed or cold-exposed male *Sphk1*^+/+^ or *Sphk1*^−/−^ mice were homogenized in lysis buffer containing 50 mM Tris-HCl (pH 7.4), 150 mM NaCl, 10% glycerol, 0.05% Triton X-100, 1 mM DTT, 2 mM Na_3_VO_4_, 10 mM NaF, 10 mM β-glycerophosphate, and 1 mM EDTA and centrifuged at 13,000 *g* for 15 min at 4°C. The supernatant was collected and centrifuged again under the same conditions to completely remove tissue debris and the fat cake. The protein content was determined in the supernatant using a protein quantification kit (Cat# PQ01, Dojindo, Kumamoto, Japan). Assay mixtures consisting of 50 mM Tris-HCl (pH 7.4), 1 mM ATP, 5 μM nitrobenzoxadiazole (NBD)-sphingosine, 15 mM MgCl_2_, 10 mM KCl, 50 mM 4-DP (Cat#D0501-1G, Sigma-Aldrich, Merck, MO), and 40 μg of tissue lysate were incubated at 37°C for 2 h. Enzyme reactions were terminated by addition of an equal volume of 1 M KPO_4_ (pH 8.5) and five volumes of chloroform/methanol (2:1, v/v). Samples were vigorously vortexed and centrifuged at 12,000 *g* for 5 min at 4°C. A portion of the upper aqueous layer was mixed with an equal volume of DMSO and fluorescence was determined using Fluoroskan Ascent FL with excitation at 485 nm and emission at 538 nm. The values of the unincubated samples were subtracted from those of the incubated samples. The subtracted values were normalized using the value of BAT (23°C) and expressed as SphK activity over the normalized values of BAT (23°C).

### Subcellular fractionation

Frozen BAT from 23°C-housed male *Sphk1*^+/+^ mice were homogenized in hypotonic buffer comprising 10 mM KCl, 1.5 mM MgCl_2_, 250 mM sucrose, protease inhibitor cocktail, and phosphatase inhibitor cocktail (1 mM Na_3_VO_4_, 10 mM β-glycerophosphate, and 10 mM NaF) in 10 mM Hepes (pH 7.5). Homogenates were incubated for 20 min on ice and then filtered using a 40 μm diameter cell strainer and centrifuged at 500 *g* for 5 min at 4°C. The pellets were washed twice with hypotonic buffer and lysed with RIPA buffer (hereafter termed the nuclear fraction). The supernatant was centrifuged at 11,000 *g* for 15 min at 4°C to remove the mitochondria and the supernatants were collected avoiding the fat cake. The supernatants were further centrifuged at 16,000 *g* for 15 min at 4°C and the resultant supernatants were collected (hereafter termed the cytoplasmic fraction). The protein content of the nuclear and cytoplasmic fractions was determined using the BCA protein assay kit and 0.3–15 μg protein was used for Western blot analysis.

The Minute lysosome isolation kit (Cat# LY-034, Invent Biotechnologies, MN) was used for lysosome enrichment. Lysosome enrichment was performed as per the manufacturer’s instruction. Pellets (at 2,000 *g*) and supernatants (at 16,000 *g*) obtained during the purification process were used as the nuclear and cytoplasmic fractions, respectively, for Western blotting. Approximately 80 μg (lysosome-rich fraction), 300 μg (nuclear fraction), and 1,500 μg proteins (cytoplasm fraction) were obtained from each BAT, and 3 μg proteins were loaded to each lane for Western blot of SphK1.

### Isolation, culture, and differentiation of stromal vascular cells

Stromal vascular cells (SVCs) from BAT of *Sphk1*^+/+^ and *Sphk1*^−/−^ mice were prepared according to previous reported protocol (24) with some modifications. In brief, BAT was dissected from male 3-week-old mice, washed with PBS, and digested in 5 ml of PBS containing 5.5 units/ml dispase II from *Bacillus polymyxa* (Roche), 0.8 mg/ml type I collagenase (Worthington, NJ), and 10 mM CaCl_2_ at 37°C under constant shaking for 90 min. Digestion was stopped by the addition of complete medium. After filtration with 100-μm diameter of cell strainer, filtrate was centrifuged for 5 min at 200 *g*. The cell pellet containing brown preadipocytes was washed twice with PBS, resuspended in complete medium, seeded on a 10-cm cell culture dish, and cultured at 37°C in a humidified atmosphere of 10% CO_2_. After 6 h, the cell culture dish was washed twice with PBS to remove unattached cells, and attached SVCs were maintained in complete medium comprising advanced DMEM/F12 supplemented with 10% FBS, 1% GlutaMAX, and 100 U/ml penicillin and 100 μg/ml streptomycin until confluent. Differentiation was induced by incubating the SVCs with induction medium (complete medium supplemented with 5 μg/ml insulin, 0.5 μM rosiglitazone, 1 nM 3,3′5-triiodo-L-thyronine, 125 μM indomethacin, 0.5 mM 3-isobutyl-1-methylxanthine, and 2 μg/ml dexamethasone). After two days, SVCs were seeded on 12-well plates or 35-mm type I collagen (Nitta gelatin, Osaka, Japan)-coated glass bottomed dishes and cultured in maintenance medium (complete medium supplemented with 1 nM 3,3′5-triiodo-L-thyronine, 5 μg/ml insulin, and 1 μM rosiglitazone). The culture medium was then changed to maintenance medium every other day until full differentiation of brown adipocytes was achieved (day 6–8).

### Analysis of NBD-sphingosine metabolism in brown adipocytes

Brown adipocytes were preincubated in maintenance medium with or without 10 μM PF-543 (Cat# 17034, Cayman, MI) or 100 μM 4-DP for 3 h. Culture media were then changed to maintenance medium containing 250 nM NBD-sphingosine (Cat# 810205X, Avanti Polar Lipids) with or without 10 μM PF-543 or 100 μM 4-DP and incubated for 1 h. Cells were stained with LysoTracker-Red (Cat# L7528, Thermo Fisher Scientific) and DAPI and imaged using confocal microscopy as described in above (see “Immunofluorescence staining”). For the analysis of NBD-sphingosine metabolites in lysosome, lysosome was enriched with Minute lysosome isolation kit. Lipids of lysosomal fractions were extracted under acidic conditions. All lipid extract of the lysosome-rich fraction (corresponding with about 4 μg protein) was applied to silica gel of TLC plate (Cat# 1.05721.0001, Merck Millipore, Darmstadt, Germany) using chloroform/methanol/28% ammonia (60:35:8, v/v) as the solvent system. Fluorescence of NBD-sphingosine metabolites were detected using a DigiPrint Doc DP-G16 TFT (BioTools) equipped with a digital camera (Powershot G16, Canon) with an LED excitation source at 470 nm. The protein content of the delipidated lysosome-rich fraction was measured using the BCA protein assay kit.

### Counting of active lysosomes in brown adipocytes

Primary brown adipocytes were incubated in maintenance medium in the presence or absence of 1 μM forskolin (Cat# 11018, Cayman) for 5.5 and 23.5 h, and then BODIPY 493/503 (4 μg/ml) was added directly to cells and incubated for 30 min. Medium was changed to maintenance medium containing LysoTracker–Red or DQ-Red BSA (Cat# D12051, Thermo Fisher Scientific) and incubated for 30 min. Cells were fixed with 4% PFA, stained with DAPI, and imaged by confocal microscopy as described above (see “Immunofluorescence staining”).

The expression vector of FLAG-tagged wild-type SphK1 (SphK1^WT^-FLAG) and catalytically inactive mutant SphK1 (SphK1^G82D^-FLAG) were kindly provided by Dr Dagmar Meyer zu Heringdorf and Dr Stuart M. Pitson, respectively. For the expression vector for monomeric GFP-tagged SphK1^WT^ (SphK1^WT^-GFP) and GFP-tagged G82D-SphK1 (SphK1^G82D^-GFP), human SphK1^WT^ and SphK1^G82D^ cording regions were amplified by PCR using PrimeSTAR Max DNA Polymerase (Cat# R045A, Takara Bio Inc., Shiga, Japan). Subcloning of the PCR products into pAcGFP1-N In-Fusion Ready vector (Cat# Z2501N, Clontech-Takara Bio Inc.) was performed using the In-Fusion HD Cloning kit (Cat# Z9633N, Clontech-Takara Bio Inc.) according to the manufacturer’s instruction. The constructed vectors were sequenced to verify the desired structure.

A rescue experiment was performed by transfecting SphK1^WT^-GFP, SphK1^G82D^-GFP, or GFP empty vector (pAcGFP1-N) into brown adipocytes using Lipofectamine 3000 reagent (Cat# L300001, Thermo Fisher Scientific). After 24 h, cells were stained with LysoTracker and DAPI and imaged by confocal microscopy.

### Live-cell imaging of brown adipocytes

Primary brown adipocytes were transfected with pLAMP1-mCherry (gifted by Amy Palmer, addgene plasmid #45147; http://n2t.net/addgene:45147; RRID:Addgene_45147, Addgene (25)) using Superfect transfection reagent (Cat# 301305, Qiagen) and incubated for 3 h. Medium was changed to maintenance medium and further incubated overnight. Cells were incubated with or without 10 μM PF-543 or 100 μM 4-DP for 3 h and then stained with BODIPY 493/503. Lysosomal motility was visualized using time-lapse imaging systems. During imaging, the growth chamber was maintained at 37°C with 5% CO_2_ saturation. Time-lapse images were taken at 10-s intervals using Andor Fusion software and movies were prepared at a frequency of 50 flames/s using Imaris software (version 9.1.2, RRID:SCR_007370, Bitplane; Oxford Instruments, Abingdon, UK).

### Acid esterase activity assay of brown adipocytes

Acid esterase activity was estimated using the fluorogenic substrate 4-methyl-umbelliferyl-palmitate as described (26) with some modifications. Primary brown adipocytes were washed twice with ice cold PBS and scraped in 200 mM sodium phosphate buffer (pH 6.8) containing 0.5% NP-40, 0.02% sodium azide, 10 mM dithiothreitol, and 1 mM EDTA. After sonication, the homogenate was centrifuged at 10,000 *g* for 30 min at 4°C. Collected supernatant was centrifuged at 10,000 *g* for 15 min at 4°C. The protein content of supernatant was determined by protein quantification kit (Dojindo). Assay mixtures containing 160 μM egg yolk phosphatidylcholine, 100 μM 4-methyl-umbelliferyl-palmitate, 0.6 mM taurocholate, and 20 μg of cell lysate in 125 mM sodium acetate (pH 4.5) were incubated at 37°C for 1 h. The reaction was stopped by the addition of 100 μl of 0.75 M Tris (pH 11). Relative fluorescence units were determined using a Fluoroskan Ascent FL with excitation at 355 nm and emission at 460 nm.

### H&E staining

Mice were intracardially perfused with PBS and 4% PFA. BAT was then dissected from the interscapular regions and fixed with 4% glutaraldehyde in PBS overnight at 4°C. BAT was embedded in paraffin and stained with H&E.

### TG quantification

For TG determination, the manufacturer’s protocol of AdipoRed assay reagent (Cat# PT-7009, Lonza Bioscience, Basel, Switzerland) was modified (hereafter referred to as modified AdipoRed assay). Fluorescence intensity dose-dependently increased by mixing TG dissolved in isopropanol and 2.5% AdipoRed, and a linear concentration range was obtained from 0.3 to 50 μg/ml triolein (Cat# 207-18201, FUJIFILM Wako Pure Chemical) (*r*^2^ = 0.997). Fluorescence intensity was not affected by the presence of oleic acid and phospholipids. Although cholesterol oleate and free cholesterol also increased fluorescence intensities, these values were approximately 40% and 85% lower, respectively, compared with that of triolein. In addition, most of the neutral lipid present in BAT was TG (checked by TLC), indicating that cholesterol ester and free cholesterol have little influence on TG quantification of BAT. We also confirmed that the TG content quantified using modified AdipoRed assay was comparable to TG determination using enzymatic kit (LabAssay Triglyceride kit).

Frozen BAT depots (around 15 mg) were homogenized in 1 ml of acetone for 2 min using TissueLyser LT (Qiagen) and zirconia beads. Homogenates were centrifuged at 10,000 *g* for 10 min at 4°C and the supernatants were transferred to 1.5-ml microcentrifuge tubes. Aliquots of the supernatants were collected and acetone was evaporated under a stream of nitrogen gas. Lipid resides were redissolved in 100 μl of isopropanol, and 10 μl of lipid extract and 190 μl of 2.5% AdipoRed solution (diluted in PBS) were mixed in 1.5-ml microcentrifuge tubes. After 10 min of incubation at room temperature, the mixtures were transferred into 96-well plate. A solution of 5 mg/ml triolein in isopropanol was used as a TG standard. Fluorescence was determined using a Fluoroskan Ascent FL with excitation at 540 nm and emission at 590 nm. Protein was measured in tissue pellets dried and lysed in 0.3 M NaOH/0.1% SDS at 50°C using the BCA protein assay kit.

TG was measured in brown adipocytes incubated in maintenance medium containing chloroquine (Cat# SIH-405-1G, StressMarq Biosciences, Victoria, British Columbia) for 24 h. Cells were then washed and harvested with 1 ml of isopropanol. After sonication, the homogenate was centrifuged at 10,000 *g* for 5 min at room temperature, and 10 μl of supernatant was used for modified AdipoRed assay. The pellet was dried and lysed with 0.3 M NaOH/0.1% SDS at 50°C and the protein content was measured using the BCA protein assay kit.

### TG hydrolase activity assay

Fresh BAT from 23°C-housed or cold-exposed male *Sphk1*^+/+^ and *Sphk1*^−/−^ mice was homogenized in 100 mM sodium phosphate buffer (pH 7.0) containing 0.5% NP-40, 0.02% sodium azide, 10 mM DTT, and 1 mM EDTA and centrifuged at 10,000 *g* for 30 min at 4°C. The supernatant was collected and centrifuged at 10,000 *g* for 15 min at 4°C. The protein content of the supernatant was measured using a protein quantification kit (Dojindo). Assay mixtures containing 2% BSA, 190 μM triolein, 0.025% Triton-X100, and 40 μg of tissue lysate in 100 mM sodium phosphate buffer (pH 7.0) were incubated at 37°C for 1 h. The NEFA content of the assay mixtures were measured using a LabAssay NEFA kit (FUJIFILM Wako Pure Chemical). The values of the unincubated samples were subtracted from those of the incubated samples, and the subtracted values were expressed as TG hydrolase activity.

### Electron microscopy

Mice were intracardially perfused with PBS and 2% PFA plus 2% glutaraldehyde in 30 mM Hepes buffer (pH 7.4). BAT was then dissected from the interscapular regions and fixed with 2% PFA plus 2% glutaraldehyde in 30 mM Hepes buffer (pH 7.4) overnight at 4°C. BAT was postfixed in 2% osmium tetraoxide for 90 min at 4°C and then dehydrated using a graded ethanol series and embedded in epoxy resin. Ultrathin sections were stained with 1% uranyl acetate for 30 min followed by Reynolds lead citrate for 5 min and then observed using a Hitach H-7650 electron microscope (Hitachi, Tokyo, Japan).

### Lipolysis in primary brown adipocytes

Lipolysis was measured in brown adipocytes as previously described with minor modification (27). SVCs (day 6) were used to induce cell differentiation. Brown adipocytes were preincubated in DMEM containing 2% BSA and 4.8 μM triacsin C (Cat# ab141888, Abcam) at 37°C for 60 min in a humidified atmosphere of 10% CO_2_ then incubated in DMEM containing 2% BSA and 4.8 μM triacsin C in the presence or absence of noradrenaline (Cat# 16673, Cayman), forskolin, S1P (Cat# 62570, Cayman), and S1P receptor-specific agonists (S1PR_1_: SEW2871 Cat# 10006440, S1PR_2_: CYM5520 Cat# 17638, and S1PR_3_: CYM5541 Cat# 15190, all Cayman) at 37°C until 4 h. The NEFA contents in medium were measured using a LabAssay NEFA kit, and protein contents were measured as described in TG quantification.

### Antibodies for immunoblotting

Antibodies used for immunoblotting were as follows: anti-SPHK1 (1:1,000) (rabbit polyclonal, kindly donated by Dr Yoshiko Banno (28)), anti-SPHK2 (1:1,000) (Cat# 17096-1-AP, RRID: AB_10598479, Proteintech), anti-lysosomal-associated membrane protein 1 (LAMP1) (1:1,000) (Cat# 121601, RRID:AB_572020, BioLegend), anti-UCP1 (1:3,000) (Cat# ab10983, RRID:AB_2241462, Abcam), anti-OXPHOS cocktail (1:2,000) (Cat# ab110413, RRID:AB_2629281, Abcam), anti-transcription factor EB (TFEB) (1:1,000) (Cat# A303-673A, RRID:AB_11204751, Bethyl Laboratories), anti-phospho-4EBP1 (1:1,000) (Thr37/46, Cat# 2855P, RRID:AB_560835, Cell Signaling Technology), anti-adipose triglyceride lipase (ATGL) (1:1,000) (Cat# 2138, RRID:AB_2167955, Cell Signaling Technology), anti-phospho-hormone sensitive lipase (HSL) (1:1,000) (Ser563, Cat# 4139, RRID:AB_2135495, Cell Signaling Technology), anti-Histone H3 (1:2,000) (Cat# 819411, RRID:AB_2820127, BioLegend), anti-GAPDH (1:1,000) (Cat# 016-25523, RRID:AB_2814991, FUJIFILM Wako Pure Chemical), and anti-β-actin (1:1,000) (Cat# 281-98721, FUJIFILM Wako Pure Chemical).

### Antibodies for immunostaining

Antibodies used for immunostaining were as follows: anti-SPHK1 (1:1,000) (rabbit polyclonal, kindly donated by Dr Yoshiko Banno), anti-LAMP1 (1:400) (Cat# 121601, RRID:AB_572020, BioLegend), anti-Rab7 (1:400) (Cat# R8779, RRID:AB_609910, Sigma-Aldrich), anti-LAMP2 (1:400) (Cat# 1921-01, AB_2795535, SouthernBiotech), anti-LC3 (1:200) (Cat# TA803577; RRID:AB_2626908, Origene), anti-CD63 (1:400) (Cat# 143901, RRID:AB_11203908, BioLegend), anti-ATPB (1:1,000) (Cat# ab14730, RRID: AB_301438, Abcam), and anti-TFEB (1:400) (Cat# NBP1-67872, RRID:AB_11043070, Novus).

### Image analysis

All images were analyzed using Image-J/Fiji software (29) (version 2.1.0/1.53c, RRID:SCR_002285). Colocalization analysis was performed using Fiji’s plugin Coloc 2. Quantification of vesicle number (Fig. 3D) was calculated by dividing the vesicle numbers in each frame by the number of nuclei in same frame and expressed as the relative value to BAT from 23°C-housed *Sphk1*^+/+^ mice. The number of vesicles in each cell were counted (Fig. 3E, F, H). Lipid droplet areas in BODIPY - or HE-stained BAT were measured using the MRI Lipid Droplets tool (http://dev.mri.cnrs.fr/projects/imagej-macros/wiki/Lipid_Droplets_Tool) (30), which is a macro of Image-J/Fiji software (Fig. 5B, C).

**Fig. 1:**
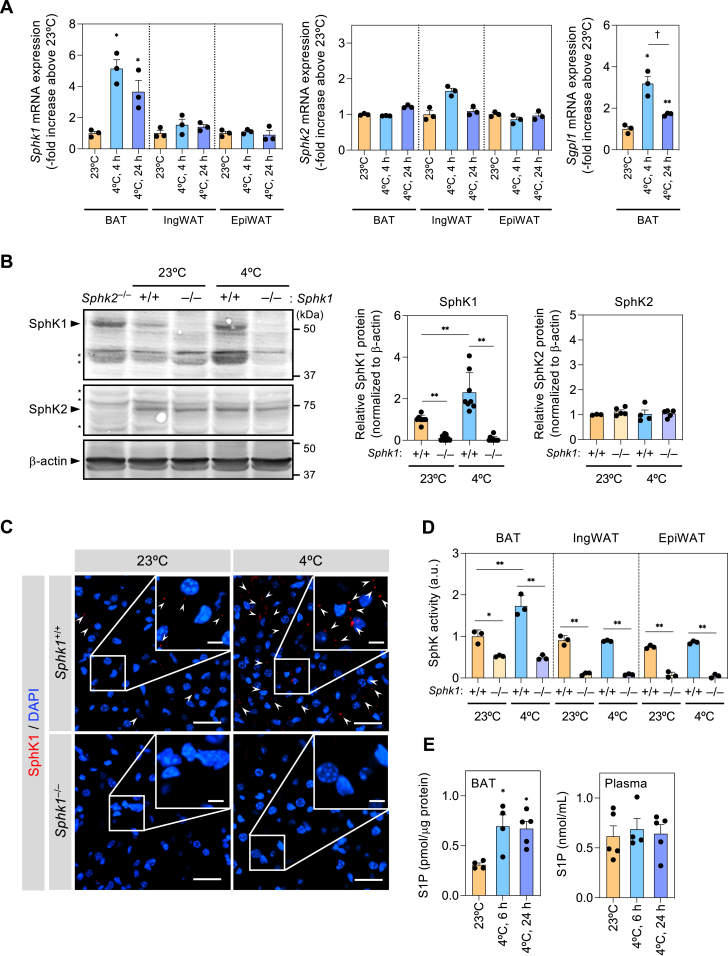
**Upregulation of SphK1 expression in BAT in response to cold exposure.** A: mRNA expression of *Sphk1*, *Sphk2*, and *Sgpl1* in BAT, inguinal WAT, and epididymal WAT from mice housed at 23°C and exposed to cold conditions (4°C) (n = 3 mice per group). Data represent the mean ± SEM and analyzed by one-way ANOVA with Tukey’s post hoc test in each adipose tissue group (∗*P* < 0.05, ∗∗*P* < 0.01 vs. 23°C, ^†^*P* < 0.05). B: Representative Western blots of SphK1 and SphK2 in BAT (left) from mice housed at 23°C and 4°C for 6 h. Quantified data from 3–10 mice per group (right) represent the mean ± SEM and analyzed by two-way ANOVA with Tukey’s post hoc test (∗∗*P* < 0.01). C: Anti-SphK1 immunofluorescent staining image of BAT from mice housed at 23°C and 4°C for 6 h. White arrowheads indicate intense SphK1-positive signals. Scale bar represents 40 μm and 10 μm in the magnified images. D: SphK activity in BAT, inguinal WAT, and epididymal WAT lysate of *Sphk1*^+/+^ and *Sphk1*^−/−^ mice (n = 3 mice per group). Data represent the mean ± SEM and analyzed by two-way ANOVA with Tukey’s post hoc test (∗*P* < 0.05, ∗∗*P* < 0.01). E: BAT and plasma S1P levels measured by liquid chromatography with tandem mass spectrometry (n = 4 ∼ 5 mice per group). Data represent the mean ± SEM and analyzed by one-way ANOVA with Tukey’s post hoc test (∗*P* < 0.05 vs. 23°C). BAT, brown adipose tissue; S1P, sphingosine 1-phosphate; SphK1, sphingosine kinase 1.

**Fig. 2:**
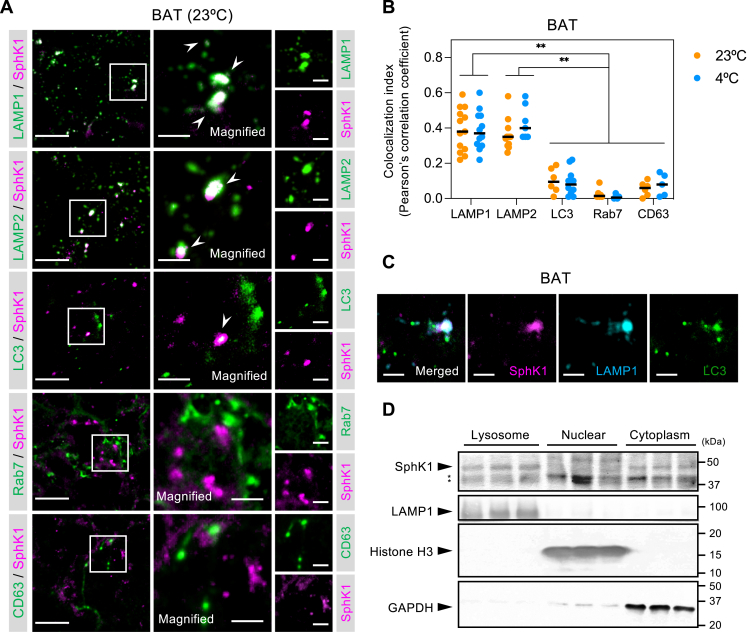
**Lysosomal localization of SphK1 in BAT.** A: Double immunofluorescent staining using anti-SphK1 and anti-organelle markers in BAT from mice housed at 23°C. Scale bar represents 20 μm and 5 μm in the magnified images. B: Colocalization indices of SphK1 to LAMP1, LAMP2, LC3, Rab7, and CD63 in BAT from mice housed at 23°C and 4°C. The data were from 4-13 images from three or more mice per group. Red bars indicate means. Two-way ANOVA with Tukey’s post hoc test was performed with comparisons (∗∗*P* < 0.01 vs. LAMP1 or LAMP2 compared with LC3, Rab7, and CD63). C: Triple immunofluorescent staining using anti-SphK1, anti-LAMP1, and anti-LC3 for analyses of the subcellular localization of SphK1 in BAT. Scale bar represents 5 μm. D: Western blot analyses of SphK1 protein in the lysosomal, nuclear, and cytoplasmic fractions from BAT. ∗ represents nonspecific bands. BAT, brown adipose tissue; LAMP, lysosomal-associated membrane protein; SphK1, sphingosine kinase 1.

**Fig. 3:**
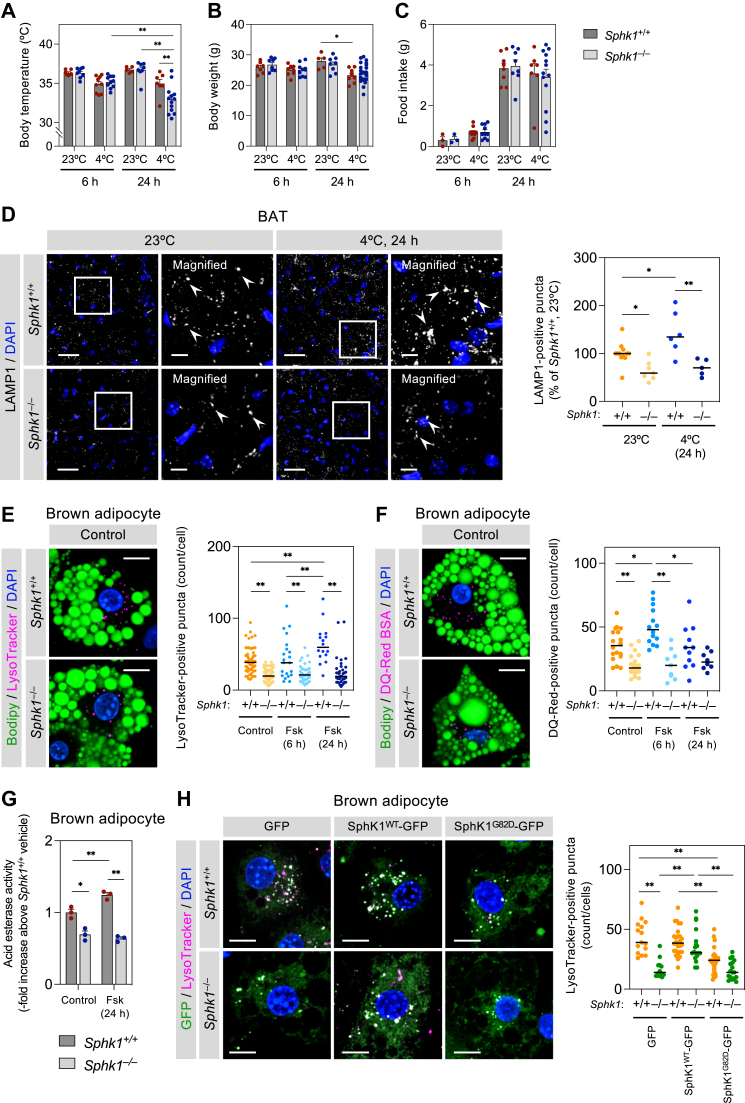
***Sphk1* deficiency decreases lysosome number and catabolic activity in brown adipocytes.** A–C: Body temperature (A), body weight (B), and food intake (C) of *Sphk1*^+/+^ and *Sphk1*^−/−^ mice housed at 23°C or 4°C (n = 3 ∼ 22 mice per group). Data represent the mean ± SEM and analyzed by two-way ANOVA with Tukey’s post hoc test (∗*P* < 0.05, ∗∗*P* < 0.01). D: Anti-LAMP1 immunofluorescent staining of BAT from mice housed at 23°C and 4°C for 24 h. The *y*-axis represents relative LAMP1-positive puncta number per cell (value of BAT from 23°C-housed *Sphk1*^+/+^ mice is expressed as 100%) (n = 5 ∼ 12 images per group from three mice). Scale bar represents 40 μm and 10 μm in the magnified images. White arrowheads indicate intense LAMP1-positive signals. Black bars indicate means. Two-way ANOVA with Tukey’s post hoc test was performed with comparisons (∗*P* < 0.05, ∗∗*P* < 0.01). E, F: Fluorescent images of *Sphk1*^+/+^ and *Sphk1*^−/−^ brown adipocytes stained with BODIPY and LysoTracker Red (E) or DQ-Red BSA (F). *Sphk1*^*+/+*^ and *Sphk1*^*−/−*^ brown adipocytes were stimulated with 1 μM forskolin (Fsk) for 6 and 24 h. Quantified data show the number of LysoTracker (n = 16 ∼ 60 cells per group) and DQ-Red BSA-positive vesicles per cell (n = 8 ∼ 19 cells per group). Scale bar represents 10 μm. Black bars indicate means. Two-way ANOVA with Tukey’s post hoc test was performed with comparisons (∗*P* < 0.05, ∗∗*P* < 0.01). G: Acid esterase activity in *Sphk1*^*+/+*^ and *Sphk1*^*−/−*^ brown adipocytes stimulated with 1 μM Fsk for 24 h (n = 3). Data represent the mean ± SEM and analyzed by two-way ANOVA with Tukey’s post hoc test (∗*P* < 0.05, ∗∗*P* < 0.01). H: Effects of SphK1^WT^-GFP and SphK1^G82D^-GFP expression on LysoTracker fluorescence. *Sphk1*^+/+^ and *Sphk1*^−/−^ brown adipocytes were transfected with SphK1^WT^-GFP, SphK1^G82D^-GFP, or GFP vectors and stained with LysoTracker (n = 12 ∼ 27 cells per group). Scale bar represents 10 μm. Black bars in graph indicate means. Two-way ANOVA with Tukey’s post hoc test was performed with comparisons (∗∗*P* < 0.01). BAT, brown adipose tissue; LAMP, lysosomal-associated membrane protein; SphK1, sphingosine kinase 1.

**Fig. 4:**
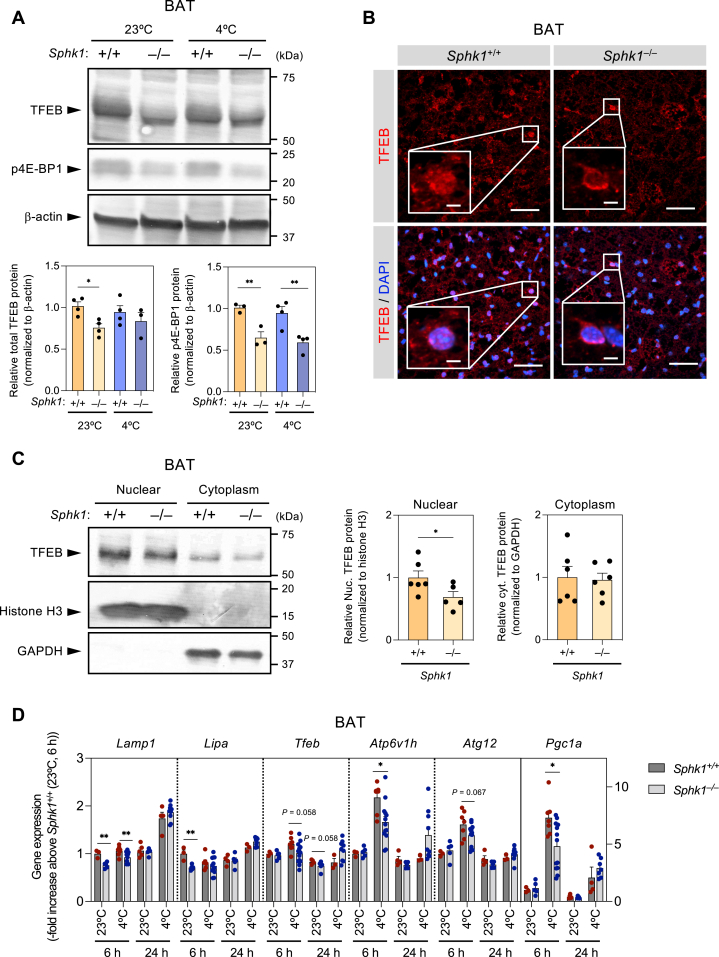
***Sphk1* deletion reduces nuclear accumulation of TFEB and expression of lysosomal genes in BAT.** A: Representative Western blots of TFEB and phosphorylated 4E-BP1 in BAT from *Sphk1*^+/+^ and *Sphk1*^−/−^ mice housed at 23°C or 4°C for 24 h (upper). Quantified data from three or four mice per group (lower). Data represent the mean ± SEM and analyzed by one-way ANOVA with Tukey’s post hoc test (∗*P* < 0.05, ∗∗*P* < 0.01). B: Anti-TFEB immunofluorescent staining of BAT from *Sphk1*^+/+^ and *Sphk1*^−/−^ mice housed at 23°C. Scale bar represents 40 μm and 5 μm in the magnified images. C: Subcellular localization of TFEB in *Sphk1*^+/+^ and *Sphk1*^−/−^ BAT (n = 5 ∼ 6 mice per group). Data represent the mean ± SEM and analyzed by Student’s *t* test for comparison between *Sphk1*^+/+^ and *Sphk1*^−/−^ groups (∗*P* < 0.05). D: mRNA expression of genes regulated by TFEB in BAT from *Sphk1*^+/+^ and *Sphk1*^−/−^ mice (n = 4 ∼ 13 mice per group). Data represent the mean ± SEM and analyzed by Student’s *t* test for comparison between *Sphk1*^+/+^ and *Sphk1*^−/−^ groups (∗*P* < 0.05, ∗∗*P* < 0.01). BAT, brown adipose tissue; SphK1, sphingosine kinase 1; TFEB, transcription factor EB.

**Fig. 5:**
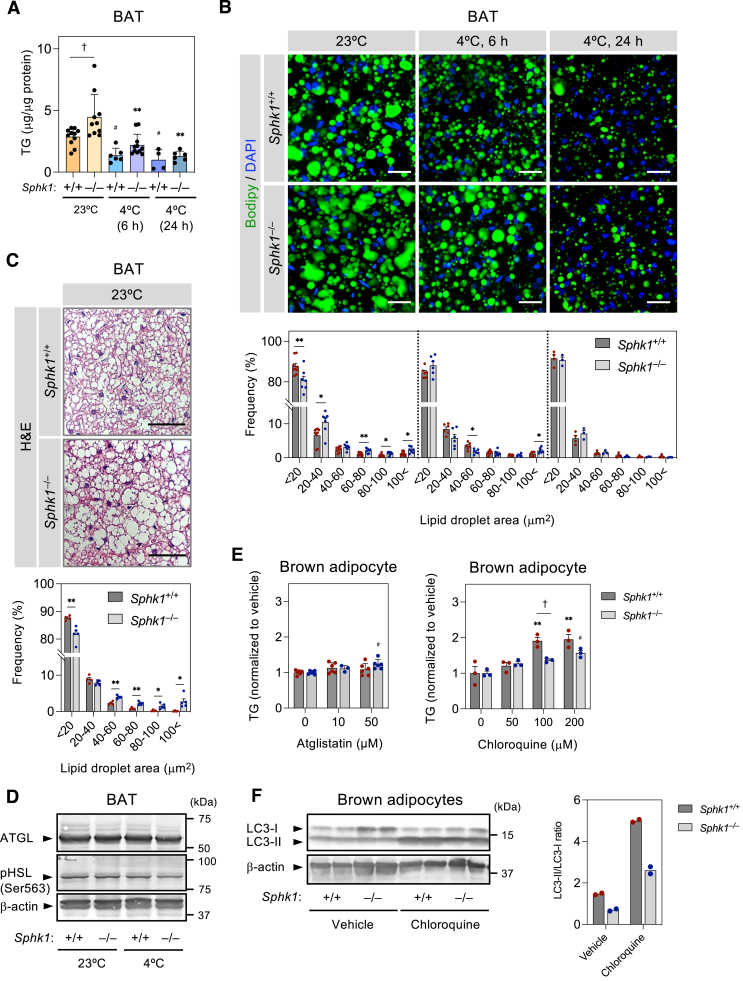
***Sphk1* deletion induces TG accumulation and large lipid droplets but does not affect cytosolic lipases in BAT.** A: Quantification of TG in BAT from *Sphk1*^+/+^ and *Sphk1*^−/−^ mice housed at 23°C and 4°C for 6 and 24 h (n = 4 ∼ 11 mice per group). Data represent the mean ± SEM and analyzed by one-way ANOVA with Tukey’s post hoc test (^#^*P* < 0.05 vs. *Sphk1*^+/+^ (23°C), ∗∗*P* < 0.01 versus *Sphk1*^−/−^ (23°C), ^†^*P* < 0.05). B: Fluorescence images in BODIPY-stained BAT from *Sphk1*^+/+^ and *Sphk1*^−/−^ mice housed at 23°C and 4°C for 6 and 24 h. Scale bar represents 40 μm. Histogram at the bottom show quantitative analysis of lipid droplet size (n = 3 ∼ 9 images per group from three or more mice). Data represent the mean ± SEM and analyzed by Student’s *t* test for comparison between *Sphk1*^+/+^ and *Sphk1*^−/−^ groups (∗*P* < 0.05, ∗∗*P* < 0.01). C: Hematoxylin and eosin (H&E) staining images of BAT from *Sphk1*^+/+^ and *Sphk1*^−/−^ mice housed at 23°C. Scale bar represents 50 μm. Histogram in the bottom panel shows quantitative analysis of lipid droplet size (n = 4 or 5 images per group from two mice). Data represent the mean ± SEM and analyzed by Student’s *t* test for comparison between *Sphk1*^+/+^ and *Sphk1*^−/−^ groups (∗*P* < 0.05, ∗∗*P* < 0.01). D: Representative Western blots of ATGL and phospho(Ser563)-HSL (activated HSL) in BAT from *Sphk1*^+/+^ and *Sphk1*^−/−^ housed at 23°C and 4°C for 6 h. E: Effects of Atglistatin or chloroquine on the TG content in brown adipocytes (n = 3 per group). Data represent the mean ± SEM and analyzed by two-way ANOVA with Tukey’s post hoc test (∗∗*P* < 0.01 vs. vehicle of *Sphk1*^+/+^, ^#^*P* < 0.05 vs. vehicle of *Sphk1*^−/−^, ^†^*P* < 0.01). F: Western blots of LC3-I and LC3-II in *Sphk1*^+/+^ and *Sphk1*^−/−^ brown adipocytes treated with or without chloroquine (left). Quantified data showed LC3-II/LC3-I ratio (right). BAT, brown adipose tissue; SphK1, sphingosine kinase 1; TG, triglyceride.

### Statistics and reproducibility

All statistical analyses were performed using GraphPad Prism 9 (version 9.5.1, GraphPad Software Inc.). The results are represented as scatter plots with median and in bar graphs as mean ± SEM. Statistical significance was evaluated using one-way ANOVA with Tukey’s post hoc test or Mann-Whitney *U* test for pairwise comparison. Differences between *Sphk1*^+/+^ and *Sphk1*^−/−^ groups and ambient temperature (23°C and 4°C) were determined using two-way ANOVA with Tukey’s post hoc test. Differences between two groups were determined by unpaired two-tailed Student’s *t* test corrected for multiple comparisons using the Holm-Šídák method. *P* < 0.05 were considered significantly different.

## Results

### Cold stimulates SphK1 expression and S1P production in BAT but not in WAT

Mice housed in a cold environment (4°C) for 4 or 24 h showed 2.8- and 12.6-fold increases in mRNA expression of classical BAT marker genes, *Ucp1* and *Pgc1a*, respectively, compared with mice housed at 23°C (supplemental Fig. S1A). This indicates that BAT was activated at 4°C. Cold exposure at 4°C for 4 and 24 h led to 5.2- and 3.7-fold increases in levels of *Sphk1* mRNA transcript, respectively, in BAT but not in inguinal WAT and epididymal WAT (Fig. 1A). In contrast to *Sphk1*, the level of *Sphk2* mRNA expression did not change in BAT, inguinal WAT, or epididymal WAT. Interestingly, mRNA levels of *Sgpl1,* encoding sphingosine 1-phosphate lyase 1, which is a key enzyme involved in S1P degradation, showed similar changes to those of *Sphk1* in BAT (Fig. 1A). In BAT, Western blot analysis showed that cold exposure increased SphK1 protein levels by 2.3-fold but did not affect SphK2 protein levels (Fig. 1B). No SphK1 protein band was detected in BAT of SphK1 KO (*Sphk1*^−/−^) mice. Immunofluorescence staining of BAT section showed that cold exposure increased SphK1-positive signals in the brown adipocytes of wild-type (*Sphk1*^+/+^) mice, whereas no obvious signal was observed in the BAT of *Sphk1*^−/−^ mice (Fig. 1C). In *Sphk1*^+/+^ mice, SphK activity of BAT but not inguinal or epididymal WAT increased in response to cold exposure (Fig. 1D). In *Sphk1*^−/−^ mice, SphK activity of BAT was almost half of that from *Sphk1*^*+/+*^ mice and failed to response to cold exposure. Cold exposure increased S1P levels in BAT but not plasma (Fig. 1E).

Primary *Sphk1*^+/+^ brown adipocytes treated with the adenylate cyclase activator forskolin (10 μM), which mimics cold and adrenergic stimulation (31, 32), showed a 2.6-fold increase in *Sphk1* mRNA levels but not *Sphk2* mRNA (supplemental Fig. S1B). Forskolin (10 μM) treatment of brown adipocytes also increased SphK activity in cell lysates (supplemental Fig. S1C). These results suggest that cold challenge induces S1P production by upregulating of SphK1 activity in brown adipocytes at least partially via mechanisms involving the cyclic AMP signaling pathway.

### Extracellular S1P and S1P agonists induce minimal stimulation of TG breakdown and thermogenic gene expression in brown adipocytes

Cold challenge (4°C) increased *S1pr1* and *S1pr3* mRNAs levels, as well as *Sphk1* and *Sgpl1* mRNA levels, in BAT (supplemental Fig. S1D). Forskolin (10 μM) treatment also increased *S1pr1* and *S1pr3* mRNA levels in brown adipocytes (supplemental Fig. S1B). Noradrenaline (10 μM) or forskolin (10 μM) treatment substantially stimulated TG breakdown, as determined by release of NEFAs in brown adipocytes. In contrast, the addition of a high concentration (10 μM) of S1P only modestly stimulated NEFA release (supplemental Fig. S2A). Furthermore, stimulation of the S1P receptor using the S1P receptor subtype-selective agonists, SEW271 (for S1PR_1_) or CYM5541 (for S1PR_3_), did not significantly increase NEFA release (supplemental Fig. S2B). In addition, S1P (1 μM) did not increase mRNA expression of the classical thermogenic genes, *Ucp1*, *Pgc1a*, *Prdm16*, and *Cidea*, in brown adipocytes (supplemental Fig. S2C). These results suggest that the effects of extracellular S1P on TG breakdown and brown adipocyte activation are minimal.

### SphK1 is localized in the lysosomes of brown adipocytes and involved in lysosomal function

SphK1 was previously reported to be mainly present in the cytoplasm and translocated to the plasma membrane in response to pharmacological stimuli (33, 34, 35, 36, 37). A recent study showed that SphK1 was also distributed in late endosomes and lysosomes (38). In the present study, immunofluorescence staining revealed that SphK1 was colocalized with the late endosome/lysosome markers LAMP1 and LAMP2 in adipocytes of BAT from mice housed at 23°C (Fig. 2A, B). However, SphK1 was not colocalized with the late endosome markers, Rab7 and CD63, suggesting that SphK1 localized with lysosomes rather than late endosomes in adipocytes of BAT. On the other hand, colocalization of SphK1 and the autophagosome/autolysosome marker, LC3, was limited, although some double-positive puncta of SphK1 and LC3 were observed that were also LAMP1-positive (Fig. 2C), implying that a portion of SphK1 is localized in autolysosomes. The lysosomal colocalization of SphK1 was similarly observed in BAT from mice housed at 4°C (Fig. 2B and supplemental Fig. S3A), indicating that lysosomal colocalization of SphK1 was not thermal stress-dependent. Furthermore, Western blot analysis of the subcellular fractions showed that SphK1 was detected in the lysosome-rich fraction as well as the cytoplasmic fraction (Fig. 2D). SphK1 protein bands in the lysosome-rich fraction were a little more dense than in the cytoplasmic fraction. However, it must be considered that 3.75% of the total lysosomal protein amounts in the lysosome-rich fraction was loaded onto SDS-PAGE, whereas only 0.2% of the total cytoplasmic protein amounts was loaded in the cytoplasmic fraction. Interestingly, SphK2 was also detected in the lysosome-rich fraction of BAT (supplemental Fig. S3B). SphK1 was also detectable in the lysosome-rich fractions of the spleen, lung, and brain (cerebral cortex) but not the kidney, liver, or testis (supplemental Fig. S3C).

We examined the effects of SphK1 inhibition on lysosomal phenotypes. The lysosomes in brown adipocytes and fibroblast-like cells isolated from the same *Sphk1*^+/+^ BAT were stained with LysoTracker and cellular sphingolipids were visualized with NBD-sphingosine labeling. In brown adipocytes, many of NBD-fluorescent puncta were LysoTracker-positive (supplemental Fig. S4A, control). Interestingly, the distribution of NBD-fluorescence strikingly differed between brown adipocytes and fibroblasts-like cells. In the fibroblasts-like cells, NBD-fluorescent puncta were fine and distributed largely in the LysoTracker-negative organelles (supplemental Fig. S4A). Furthermore, TLC analysis of the lysosome-rich fractions from brown adipocytes revealed the presence of NBD-S1P (supplemental Fig. S4B). Treatment of brown adipocytes with the SphK1-selective inhibitor, PF-543 (39), decreased the amount of NBD-S1P, whereas treatment with the Sgpl1 inhibitor 4-DP increased the amount of NBD-S1P (supplemental Fig. S4B). Importantly, PF-543 treatment led to lysosomal enlargement due to its aggregation, whereas 4-DP treatment resulted in the accumulation of smaller LysoTracker-positive puncta (supplemental Fig. S4A). Moreover, the motility of enlarged lysosomes in PF-543-treated brown adipocytes was reduced compared with that of control and 4-DP-treated cells (supplemental Videos S1–S3). These observations indicate that modulation of sphingolipid metabolism influences lysosomal morphology and motility in brown adipocytes.

### Deletion of *Sphk1* impairs cold tolerance and diminishes the cold-induced increase in lysosomes and their functional upregulation in brown adipocytes

We investigated the influence of genetic *Sphk1* deletion on lysosomal functions and accompanying BAT activity. Under cold conditions, the rectal temperature of *Sphk1*^−/−^ mice at 24 h was slightly but significantly decreased compared with *Sphk1*^+/+^ mice (*P* = 0.0097), although these differences were not observed at 6 h (Fig. 3A). A significant decrease in body weight at 24 h of cold exposure was observed in *Sphk1*^+/+^ mice but not *Sphk1*^−/−^ mice (Fig. 3B). There were no differences in the amount of food intake and serum lipid levels (TG, total cholesterol, and NEFA) between *Sphk1*^+/+^ and *Sphk1*^−/−^ mice (Fig. 3C and supplemental Fig. S5A). Furthermore, no obvious change was observed in mRNA expression levels of the fatty acid transporters, *Fabp4* and *Cd36*, and glucose transporters, *Glut1* and *Glut4* (supplemental Fig. S5B). The mitochondrial protein UCP1 in *Sphk1*^+/+^ mice was not increased by cold exposure (supplemental Fig. S5C), although its mRNA level was elevated by cold (supplemental Fig. S1A). In addition, UCP1 protein level was not different between *Sphk1*^+/+^ and *Sphk1*^−/−^ mice. Similar to UCP1, the level of mtDNA as expressed as mtDNA to nuclear DNA ratio and the protein levels of mitochondrial respiratory complexes were also comparable between *Sphk1*^+/+^ and *Sphk1*^−/−^ mice and unaltered by cold (supplemental Fig. S5D, E). Furthermore, the morphology and distribution pattern of mitochondria of *Sphk1*^−/−^ brown adipocytes were similar to those of *Sphk1*^+/+^ brown adipocytes (supplemental Fig. S5F, G).

Since we observed dominant localization of SphK1 in the lysosomes in brown adipocytes, we evaluated the lysosomes in BAT from *Sphk1*^+/+^ and *Sphk1*^−/−^ mice housed at 23°C and 4°C. Immunofluorescence staining showed a reduction in LAMP1-positive puncta in the BAT of *Sphk1*^−/−^ mice at 23°C compared with *Sphk1*^+/+^ mice (Fig. 3D). Cold exposure increased LAMP1-positive puncta in *Sphk1*^+/+^ mice via stimulation of lysosomal biogenesis (40). In contrast, cold exposure did not increase LAMP1-positive puncta in *Sphk1*^−/−^ mice. Similar to the effects of cold exposure in BAT, forskolin (1 μM) stimulation induced an increase in LysoTracker-positive puncta in *Sphk1*^+/+^ brown adipocytes (Fig. 3E). The number of LysoTracker-positive puncta in resting *Sphk1*^−/−^ brown adipocytes were also reduced compared with those in *Sphk1*^*+/+*^ brown adipocytes, and forskolin (1 μM) failed to increase the number of LysoTracker-positive puncta in *Sphk1*^−/−^ brown adipocytes at 24 h, which was in contrast to *Sphk1*^+/+^ brown adipocytes (Fig. 3E). *Sphk1*^*+/+*^ brown adipocytes showed a rise of lysosomal proteolytic activity in response to forskolin, as visualized using DQ-Red BSA staining (Fig. 3F). In contrast, *Sphk1*^*−/−*^ brown adipocytes, which had a lower resting proteolytic activity, showed no upregulation of lysosomal proteolytic activity in response to forskoline. *Sphk1*^*−/−*^ brown adipocytes also exhibited reduced acid esterase activity compared with *Sphk1*^+/+^ brown adipocytes (Fig. 3G). To confirm the observed phenotypes resulting from the loss of SphK1 activity, GFP-tagged wild-type SphK1 (SphK1^WT^-GFP) or catalytically inactive SphK1 (SphK1^G82D^-GFP) were transiently expressed in *Sphk1*^−/−^ brown adipocytes. The expression of SphK1^WT^-GFP in *Sphk1*^−/−^ brown adipocytes increased SphK activity up to the level of nontransfected *Sphk1*^+/+^ brown adipocytes (supplemental Fig. S6) and restored the number of LysoTracker-positive puncta to a similar level as that seen in *Sphk1*^+/+^ brown adipocytes (Fig. 3H). In contrast, the expression of SphK1^G82D^-GFP in *Sphk1*^−/−^ brown adipocytes failed to rescue the decreased number of LysoTracker-positive puncta (Fig. 3H). In *Sphk1*^+/+^ brown adipocytes transfected with SphK1^G82D^-GFP, a slight decrease of LysoTracker-positive puncta was observed, which might be due to a dominant-negative effect of SphK1^G82D^. These results suggest that the reduced functional lysosomes in *Sphk1*^−/−^ brown adipocytes is due to loss of the enzymatic activity of SphK1. Furthermore, *Sphk1*^−/−^ brown adipocytes also showed reduced lysosomal mobility compared with *Sphk1*^+/+^ brown adipocytes, as visualized using LAMP1-mCherry fluorescence (supplemental Videos S4 and S5).

We studied the mechanisms underlying the impairment of lysosomal biogenesis in *Sphk1*^−/−^ brown adipocytes. TFEB is the master transcriptional regulator essential for lysosomal biogenesis and its functional integrity (41, 42) and its phosphorylation status regulates its activity. Dephosphorylated TFEB is active from and translocates into the nucleus to stimulate transcription of its target genes. Western blot analysis revealed that the intensity of the upper portion in the TFEB band, which corresponding to phosphorylated from of TFEB, was fainter in the BAT of *Sphk1*^−/−^ mice housed at 23°C compared with *Sphk1*^+/+^ mice housed at 23°C (Fig. 4A). This was accompanied by a decrease in the amount of total TFEB (Fig. 4A). Cold exposure did not affect the phosphorylation status of TFEB in either *Sphk1*^+/+^ or *Sphk1*^−/−^ mice. Phosphorylation of TFEB is mainly controlled by mammalian target of rapamycin complex 1 (mTORC1) (43). In *Sphk1*^−/−^ mice, the protein kinase activity of mTORC1 in BAT was decreased compared with that of *Sphk1*^+/+^ mice, as evaluated by phosphorylation of endogenous mTORC1 substrate, 4E-BP1 (Fig. 4A), which was consistent with the TFEB phosphorylation status. However, anti-TFEB-immunofluorescence staining of BAT showed that the central area inside the nucleus was positive for TFEB in *Sphk1*^+/+^ mice, whereas TFEB signal in *Sphk1*^−/−^ mice was localized around the nuclear membrane but not inside the nucleus (Fig. 4B). Moreover, Western blot analysis showed that the amount of TFEB protein in nuclear fraction of BAT was decreased by 30% in *Sphk1*^−/−^ mice compared with *Sphk1*^+/+^ mice (Fig. 4C), which was consistent with our morphological findings. In BAT, mRNA levels of the TFEB target genes, *Lamp1*, *Lipa*, *Atp6v1h*, *Atg12, Pgc1a*, and *Tfeb*, were decreased by 15%–30% in *Sphk1*^−/−^ mice compared with *Sphk1*^+/+^ mice (Fig. 4D). Therefore, it is possible that SphK1 is involved in the regulation of TFEB activity via a mechanism that is not directly dependent on TFEB phosphorylation.

### *Sphk1* deletion leads to accumulation of TG and large lipid droplets but does not affect cytosolic lipases in BAT

Lysosomes are involved in autophagic lipid droplet breakdown (lipophagy) catalyzed by lysosomal acid lipase; therefore, the decrease of functionally intact lysosomes was expected to affect metabolism of lipid droplets in *Sphk1*^−/−^ BAT. Lipophagy and lipid droplet breakdown by cytosolic lipases cooperatively contribute toward total TG breakdown in brown adipocytes (40). Therefore, we examined the influence of *Sphk1* deletion on total TG breakdown in BAT. The TG content of BAT in *Sphk1*^−/−^ mice housed at 23°C was around 1.7-fold higher than that of *Sphk1*^+/+^ mice (Fig. 5A). Although cold exposure decreased the TG content in the BAT of both *Sphk1*^+/+^ and *Sphk1*^−/−^ mice, the TG content in the BAT of *Sphk1*^−/−^ mice tended to be higher than in *Sphk1*^+/+^ mice after 6 h of cold exposure. In accordance with these findings, large lipid droplets in BAT were more abundant in *Sphk1*^−/−^ mice compared with *Sphk1*^+/+^ mice, particularly in mice housed at 23°C (Fig. 5B, C, and supplemental Fig. S7A). In *Sphk1*^−/−^ BAT, mRNA and protein levels of the major cytosolic lipases, ATGL and HSL, were comparable to those in *Sphk1*^+/+^ mice (Fig. 5D and supplemental Fig. S7B). TG hydrolase activity in BAT lysate at neutral pH was similar in *Sphk1*^+/+^ and *Sphk1*^−/−^ mice (supplemental Fig. S7C). We studied the effects of the ATGL inhibitor Atglistatin on TG accumulation. Atglistatin at 10 μM did not affect TG accumulation in either *Sphk1*^+/+^ or *Sphk1*^−/−^ brown adipocytes and at 50 μM, increased TG level slightly (20%) in *Sphk1*^*−/−*^ brown adipocytes but not *Sphk1*^*+/+*^ brown adipocytes (Fig. 5E). In contrast, blockage of autophagy by chloroquine, which inhibits the fusion of autophagosomes with lysosomes (44), resulted in substantial TG accumulation in *Sphk1*^+/+^ brown adipocytes (Fig. 5E). In *Sphk1*^−/−^ brown adipocytes, chloroquine was less effective for TG accumulation than *Sphk1*^+/+^ brown adipocytes (Fig. 5E). Moreover, Western blot analysis showed that chloroquine increased LC3-II in *Sphk1*^*+/+*^ and *Sphk1*^−/−^ brown adipocytes (Fig. 5F). Notably, chloroquine-induced increases of LC3-II/ LC3-I ratio was smaller in *Sphk1*^−/−^ brown adipocytes than in *Sphk1*^+/+^ adipocytes. These observations suggest the impaired autophagy flux due to *Sphk1* deficiency. Moreover, there was no difference in mRNA levels of TG synthesis-related genes in both mouse groups (supplemental Fig. S7B). Taken together, these results suggest that the greater accumulation of TG seen in the BAT of *Sphk1*^−/−^ mice is due to reduced lipophagy rather than reduced neutral lipolysis by cytosolic lipases or increased TG synthesis.

## Discussion

The present study revealed that cold exposure upregulates the expression of SphK1 but not SphK2 in the lysosomes of brown adipocytes and that SphK1 is involved in the thermogenic response, likely via lysosome-mediated TG breakdown in brown adipocytes. SphK1 is essential for cold-induced changes in the lysosomes, particularly the increase in the lysosomal number. Since both S1P deletion via pharmacological SphK1 blockage and S1P accumulation via inhibition of the S1P-degrading enzyme Sgpl1 led to marked changes in the size and number of the lysosomes in brown adipocytes, it is likely that the enzyme activity of SphK1 and the resultant concordant sphingolipid metabolism in the lysosomes play a crucial role in the integrity of the lysosomes. This action of SphK1 on the lysosomes is at least partially mediated by the effects of SphK1 on the master transcriptional factor, TFEB, which is essential for lysosomal biogenesis and its functional integrity.

The SphK1 substrate, sphingosine, is generated as an end-product of sphingolipid degradation inside the lysosomes (45) and is thought to be transferred to the outer leaflet of the lysosomal membrane, although the mechanisms by which sphingosine passes through the lysosomal membrane are not well understood (46, 47). Sphingosine is, then, converted to S1P by the action of SphK1 on the lysosomes, followed by cleavage of the generated S1P to hexadecenal and phosphoethanolamine by the action of Sgpl1. Cold exposure enhanced SphK1 expression and stimulated S1P production. At the same time, cold exposure also increased Sgpl1 expression. Short-term inhibition of SphK1 or Sgpl1 action altered the size and the number of the lysosomes, despite exerting contradictory effects on S1P levels in brown adipocytes. Therefore, the accumulation of sphingosine and/or its precursor ceramide caused by inhibition of SphK1 or Sgpl1, as well as disturbed levels of S1P itself, in the lysosomes may impair lysosomal biogenesis and maturation. The physicochemical properties of sphingosine and S1P mean that accumulation of sphingosine on outer membrane forms the positively charged domains (46), which may facilitate aggregation and fusion through electrostatic attraction between vesicles. Negatively charged S1P may interact with sphingosine electrostatically and neutralize a surface charge (48), suggesting that the imbalance of sphingosine and S1P content in the lysosomal membrane is responsible for abnormal membrane fusion and fission.

In the present study, BAT lysosomes showed relatively high levels of SphK1 among various mouse tissues examined, with the following rank order: spleen ≥ BAT ≥ lung > brain (cerebral cortex) > kidney, liver, and testis (supplemental Fig. S3C). Interestingly, the order of expression levels showed a similar trend with SphK activity in the mouse tissues (49) and *SPHK1* mRNA in human tissue (50). Organs with higher levels of lysosomal SphK1 expression and activity included those that process large amounts of lipid, such as spleen (aged cell membrane), lung (lipid surfactants), and brain (myelin sheath). In brown adipocytes, this finding supports the importance of lysosomal SphK1 in lysosome-mediated TG breakdown.

Unlike short-term inhibition of SphK1, *Sphk1* deficiency decreased the number of functionally intact lysosomes, likely at least partly due to the reduced nuclear translocation of TFEB. Impaired TFEB nuclear translocation was accompanied by downregulation of mTORC1 activity as suggested by decreased 4E-BP1 phosphorylation. Decreased mTOR activity should dephosphorylate TFEB and activate TFEB (43), which was contradictory to the present findings, suggesting that an mTORC1-independent mechanism is involved in the reduced nuclear translocation of TFEB. Although the underlying mechanisms remain unclear, the change in the lipid composition in the lysosomal membrane seen with *Sphk1* deficiency may be key point for the following reasons. (1) Intracellular sphingosine, but not S1P, is directly related to both lysosomal calcium release and refilling (47, 51, 52, 53). Lysosomal calcium content was markedly reduced by sphingosine accumulation in the lysosome membrane that was induced by *Sphk1* deficiency or inhibition (8). (2) Lysosomal calcium release induces translocation of TFEB to the nucleus, and the inactivation of lysosomal calcium releasing channels by the genetic and pharmacological technique suppressed TFEB translocation (51, 54). (3) Sphingolipids may influence nucleocytoplasmic trafficking via the importin-dependent transport system. For instance, ceramide, ceramide 1-phosphate, and sphingomyelin inhibit nuclear import, whereas S1P promotes nuclear import (55). Further studies are required to clarify the mechanisms involved in impaired nuclear translocation of TFEB.

Several studies have reported that sphingolipids, including S1P, regulate autophagy. The balance between the production and degradation of S1P may be a critical determinant in S1P-regulated autophagic mechanisms. For example, deficiency of *Sgpl1* and endoplasmic reticulum-residing S1P phosphatase augment S1P levels and induce autophagy (56, 57). *Sphk2*-deficient macrophages display defective autophagic breakdown of lipid droplets, accompanied by impaired luminal acidic environment and proteolytic activity in lysosomes (38). In addition, inactivation of SphK1 impairs autophagic flux via inhibition of autophagosome and lysosome fusion in Niemann-Pick type C patient fibroblasts (8). Moreover, genetic deletion of the autophagy components, *Atg5* and *Atg7*, and pharmacological inhibition of autophagy promote TG accumulation in BAT and liver (58, 59). These findings suggest a critical connection between sphingolipid metabolism and autophagy in TG metabolism. In the present study, *Sphk1* deletion affected lysosomal morphology and function in brown adipocytes. In addition, *Sphk1* deficiency is likely to affect the steady-state LC3 flux (supplemental Fig. S7D), thereby altering the autophagic activity. Moreover, treatment of brown adipocytes with the lysosomal inhibitor, chloroquine, resulted in TG accumulation, suggesting that lysosomes have a great impact on TG degradation in brown adipocytes. *Sphk1*^−/−^ brown adipocytes showed a less pronounced inhibition by chloroquine than *Sphk1*^+/+^ brown adipocytes, suggesting a lower capacity for lipophagy in *Sphk1*^−/−^ brown adipocytes. In addition to lipophagy, neutral lipolysis by the cytosolic lipases, ATGL and HSL, cooperatively contributes to total TG breakdown in brown adipocytes (40, 60). However, the protein levels and activities of the cytosolic lipases in *Sphk1*^−/−^ BAT did not differ from those in *Sphk1*^+/+^ BAT in mice housed at 23°C and 4°C. In addition, ATGL inhibitor was less effective on TG accumulation in brown adipocytes. Therefore, the contribution of SphK1 to neutral lipolysis appears to be minimal. At 23°C, the influence of *Sphk1* deletion in total TG breakdown may be greater in lipophagy rather than neutral lipolysis, since both lysosomal proteolytic activity and acid esterase activity of *Sphk1*^*−/−*^ brown adipocytes were lower than those of *Sphk*^*+/+*^ brown adipocytes (Fig. 3F, G). On the other hand, in cold conditions, when both lipophagy and neutral lipolysis are upregulated (40), cytosolic lipolysis may contribute to total TG breakdown to a great extent than lipophagy, and consequently, the difference in TG content between *Sphk1*^+/+^ and *Sphk1*^−/−^ BAT was reduced in the present study. Therefore, we propose that SphK1 largely contributes to total TG breakdown via lipophagy, which is mediated, at least in part, by the regulation of lysosomal biogenesis and integrity in BAT. Furthermore, SphK2 was also localized in BAT lysosomes (supplemental Fig. S3B), suggesting that both SphK1 and SphK2 coordinately may work there. Therefore, both SphK1 and SphK2 depletion in BAT lysosomes would help elucidate the role of SphKs on the lysosomal function.

Previous study showed that global *Sphk1* depletion suppressed TG accumulation in liver and hepatic steatosis on diet-induced obese mice (61). This indicates that SphK1 contributes to TG metabolism in not only BAT but also liver. However, in our experimental condition in which *Sphk1*^*−/−*^ and *Sphk1*^*+/+*^ mice were fed normal diet and nonobese, TG level in liver was comparable in both mice groups. Unlike BAT, liver contains a modest level of SphK1 in the lysosome-rich fraction (supplemental Fig. S3C). These findings suggest that, in normal diet-fed mice, the contribution of SphK1 to lipophagy in liver may be relatively smaller than brown adipocytes.

Cold exposure upregulated both S1P production and *S1pr1* and *S1pr3* transcription in BAT. A previous study showed that S1P induces TG breakdown via adenylyl cyclase-coupled S1P receptors in rat white adipocytes (62). Given these findings, we postulated that S1P contributes to TG breakdown via S1P receptor(s) for thermogenesis in BAT. In the present study, TG breakdown was stimulated by exogenous S1P via S1PR_1_ and S1PR_3_ in brown adipocytes. However, stimulation of TG breakdown was achieved using 10 μM S1P, which is considerably higher than the physiological plasma S1P concentration (around 0.5 μM) (Fig. 1E). Although such a high S1P concentration may be reached when S1P is robustly produced locally in BAT activated by cold exposure, TG breakdown via S1P is much weaker than that of the physiological stimulus noradrenaline. Therefore, extracellular S1P is thought to have only a marginal effect on TG breakdown in brown adipocytes.

The thermogenic response of *Sphk1*^−/−^ mice to 24 h of cold exposure was slightly but significantly decreased by *Sphk1* deletion. Since TFEB in brown adipocytes recently shown to contribute to thermogenesis via the promotion of mitochondrial biogenesis (63) and the suppression of mitochondrial degradation (64), the decrease in mitochondrial function was suspected to be causes of hypothermia in *Sphk1*^−/−^ mice. However, in *Sphk1*^−/−^ mice, protein levels of UCP1 and mitochondrial respiratory complexes and mitochondrial DNA and morphology in BAT were comparable to those of *Sphk1*^+/+^ mice housed at 4°C and 23°C, suggesting that the process of mitochondrial thermogenesis itself in *Sphk1*^−/−^ BAT is normal. Furthermore, circulating nutrients were sufficient to replenish BAT energy demands in *Sphk1*^−/−^ mice. It was recently reported that young lysosomal acid lipase gene-deficient (*Lal*^−/−^) mice (aged 6 weeks) showed severe hypothermia within a few hours of cold exposure (65). Young *Lal*^−/−^ mice were similar to *Sphk1*^−/−^ mice, in that lipid droplets were enlarged and mitochondrial structure was normal in BAT compared with wild-type mice (65, 66). In addition, very recently, we found that impairment of fatty acid generation and utilization in BAT results in the lower heat production capacity, even if UCP1 levels are higher (67). Considering these findings, the decreased fuel supply by impaired lysosomal activity could, at least in part, be involved in hypothermia of *Sphk1*^−/−^ mice. However, since global *Sphk1*^−/−^ mice were used in this study, the contribution of SphK1 in tissues other than BAT to cold tolerance remains to be fully explored.

## Conclusion

We demonstrated that intact SphK1 activity and the resultant sphingolipid environment play a role in the formation of functionally intact lysosomes in brown adipocytes. Disruption of sphingolipid metabolism in lysosomes led to functional deficiency of the lysosomes and an insufficient thermogenic response in brown adipocytes. Controlling lysosomal function by modulating sphingolipid metabolism in brown adipocytes may be a novel therapeutic approach to target obesity.

### Limitations of the study

In this study, we analyzed global *Sphk1*^−/−^ mice and observed that BAT and brown adipocytes isolated from *Sphk1*^−/−^ mice showed clear-cut difference compared with control mice. However, further studies using cell type-specific conditional *Sphk1*^−/−^ mice, particularly brown adipocyte-specific knockout mice, are required for understanding the influence of *Sphk1* deficiency in non-BAT tissues. We showed that *Sphk1* deficiency impairs lipophagy. It would be important to understand the precise molecular mechanisms by which the derangements of sphingolipid metabolism induced by *Sphk1* deficiency result in impaired lipophagy, for developing a new anti-obesity therapeutic strategy to target lipophagy. Finally, we also need to understand the exact roles of not only SphK1 but SphK2 in lysosomal integrity, TG breakdown, and cold tolerance. SphK2 is also localized in the lysosomes of brown adipocytes. Therefore, it is possible that deficiency of SphK1 actions could be at least in part compensated by SphK2 in *Sphk1*^−/−^ mice and that, consequently, *Sphk1*^−/−^ mice could have mild phenotypes. *Sphk1* and *Sphk2* double global knockout mice are embryonic lethal (68). Analyses of cell type-specific or conditional knockout mice of *Sphk1* and *Sphk2* would provide important information to understand a role of SphK2.

## Data Availability

Data will be made available on request.

## Supplemental data

This article includes supplemental data.

## Conflict of interest

The authors declare that they have no conflicts of interest with the contents of this article.
