# Supplementary material for: Sphingosine kinase 1 is involved in triglyceride breakdown by maintaining lysosomal integrity in brown adipocytes
Source: J Lipid Res. 2023 Sep 24;64(11):100450. doi: 10.1016/j.jlr.2023.100450 (PMC10630120; doi:10.1016/j.jlr.2023.100450)
Supplement: Supplementary Figures and Table [file mmc1.pdf]

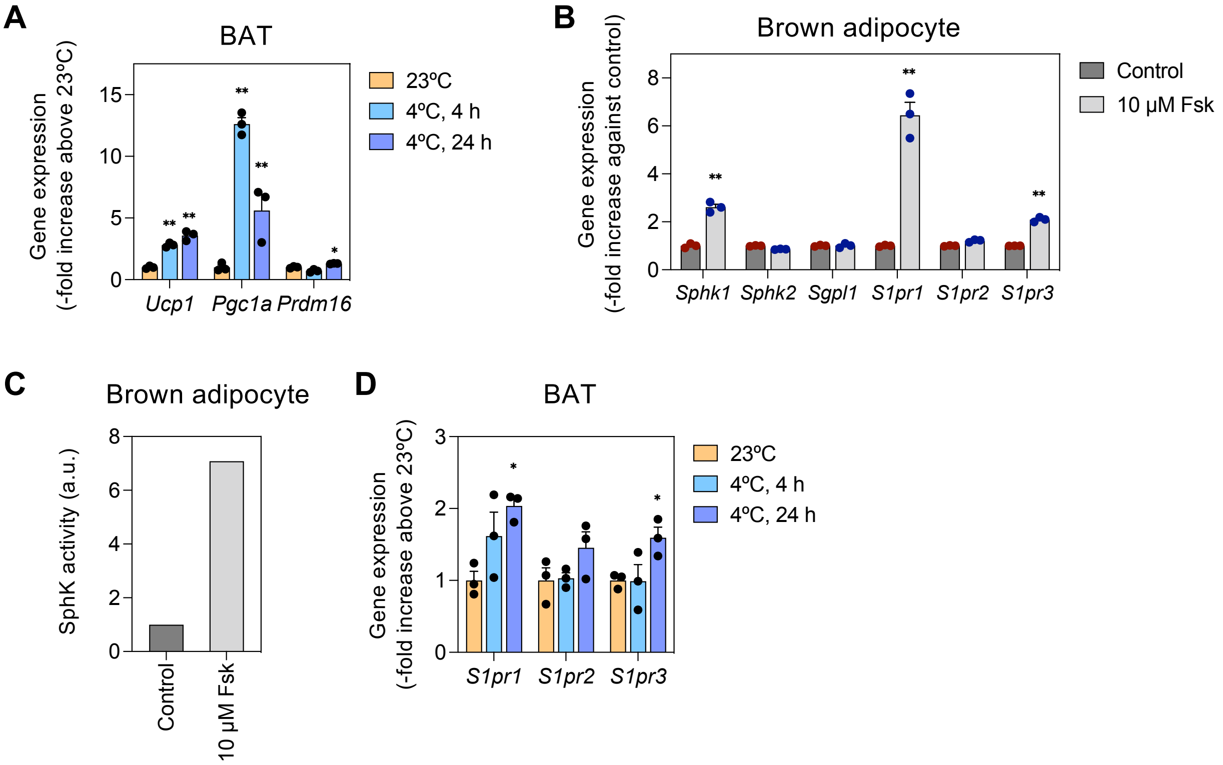

Figure S1. Upregulation of thermogenic genes, SphK1, S1P receptors, and SphK1 activity in BAT and primary brown adipocytes.

(A) mRNA expression of classical thermogenic genes in BAT (n = 3 mice per group). Data represent the mean ± SEM and analyzed by one-way ANOVA with Tukey's post hoc test ($^{**}P < 0.01$ versus 23ºC). (B) mRNA expression in brown adipocytes treated with 10 μM forskolin (Fsk). Data represent the mean ± SEM and analyzed by Student's t-test for comparison of control ($^{**}P < 0.01$). (C) SphK activity of cell lysate (20 μg of protein) prepared from brown adipocytes treated with or without 10 μM Fsk. (D) mRNA expression of S1P receptors in BAT (n = 3 mice per group). Data represent the mean ± SEM and analyzed by one-way ANOVA with Tukey's post hoc test ($^{*}P$ < 0.05 versus 23ºC).

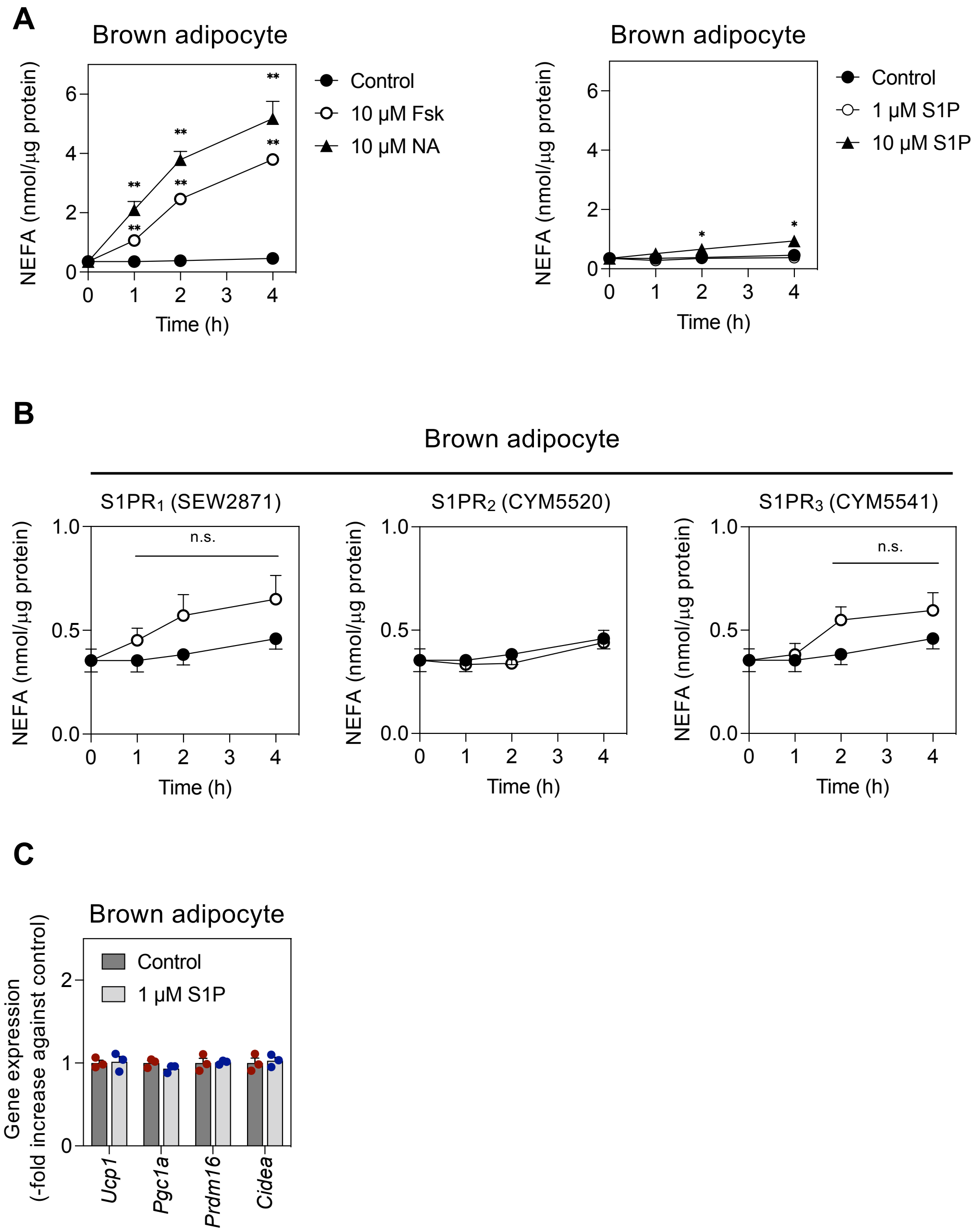

Figure S2. Effect of forskolin, noradrenaline, and exogenous S1P on lipolysis and thermogenic gene expression.

(A) and (B) Brown adipocytes were stimulated by Fsk, noradrenaline (NA), S1P, and S1P receptor subtype-selective agonists ($S1PR_1$: SEW2871, $S1PR_2$: CYM5520, and $S1PR_3$: CYM5541). Lipolysis activity was determined as released amounts of NEFA. Data represent the mean ± SEM. Dunnett's multiple comparisons test was performed with comparisons to control group ($^{*}P < 0.05$, $^{**}P < 0.01$). n.s., not significant. (C) mRNA expression of classical thermogenic genes. mRNA levels are expressed relative to *Ppia* mRNA expression in control brown adipocytes.

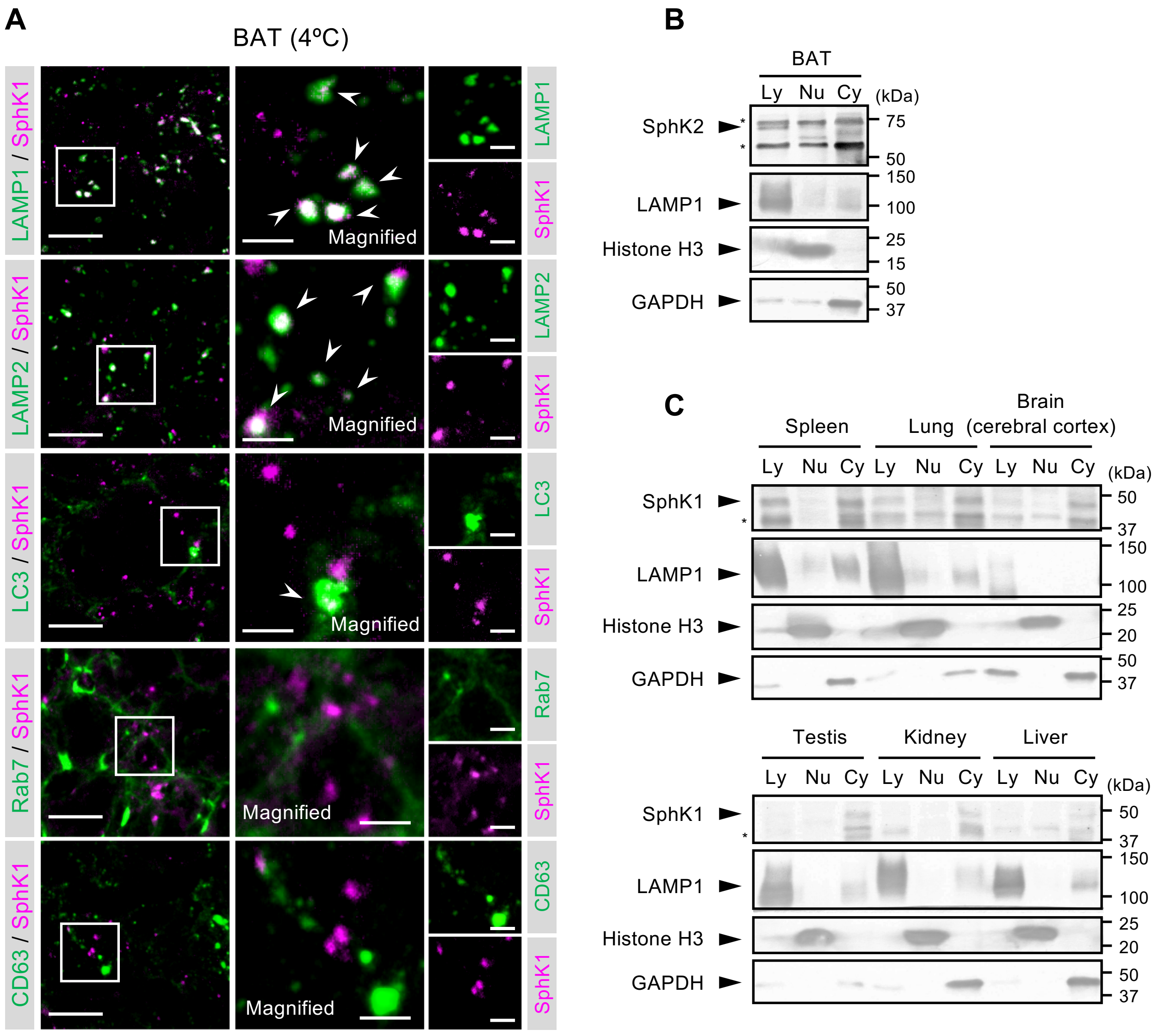

Figure S3. Lysosomal localization of SphK1 in BAT and subcellular distribution of SphK1 and SphK2 in mouse tissues.

(A) Double immunofluorescent staining using anti-SphK1 and anti-organelle markers in BAT from mice housed at 4ºC. Scale bar represents 20 µm and 5 µm in the magnified images. Colocalization indices of SphK1 to LAMP1, LAMP2, LC3, Rab7, and CD63 were shown in Figure 2B. (B) and (C) Subcellular distribution of SphK2 in BAT (B) and SphK1 in spleen, lung, brain (cerebral cortex), testis, kidney, and liver (C). Western blots of lysosomal, nuclear, and cytoplasmic fractions from various tissues. *Nonspecific bands. Ly, lysosome-rich fraction; Nu, nuclear fraction; Cy, cytoplasmic fraction.

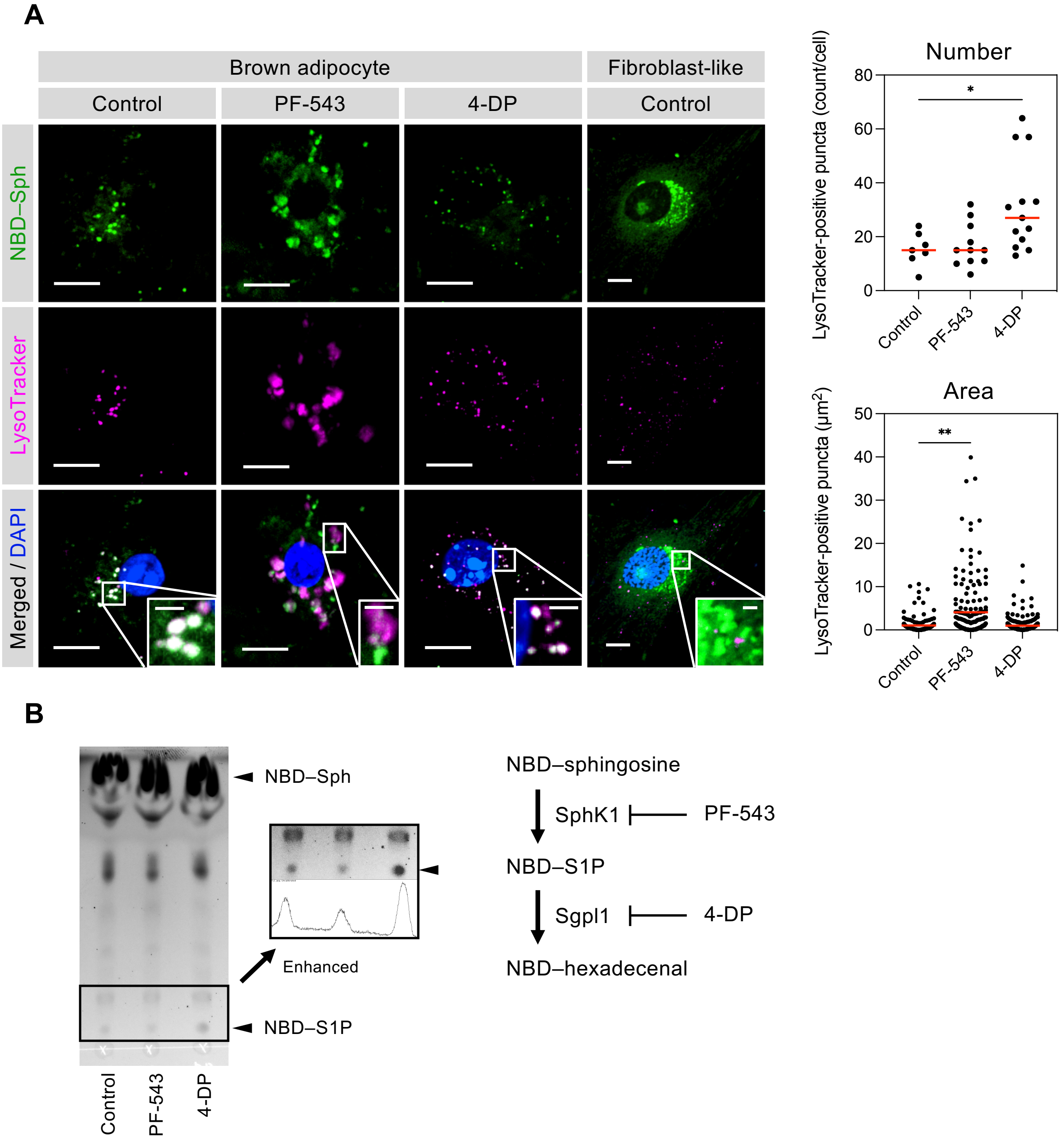

Figure S4. NBD–S1P production in the lysosomes of brown adipocytes.
(A) Representative immunofluorescence images of brown adipocytes and fibroblasts-like cells (left). Cells were treated with 250 nM NBD–sphingosine for 1 h in the presence or absence of 10 µM PF-543 (SphK1 inhibitor) or 100 µM 4-DP (Sgpl1 inhibitor). Scale bar represents 10 µm and 2 µm in the magnified images. Quantified data show the number and area of LysoTracker positive puncta (right) (n = 7~13 cells from 3 or more images per group). Red bars indicate means. Dunnett's multiple comparisons test was performed with comparisons to control group ($^{*}P < 0.05$, $^{**}P < 0.01$). (B) TLC of lipids extracted from the lysosomal fractions of brown adipocytes cultured in the same conditions as (A). Boxed regions are magnified and enhanced. Histogram shows the fluorescence intensity of NBD–S1P. Bottom scheme illustrates the NBD–sphingosine metabolic pathway and targets of PF-543 and 4-DP.

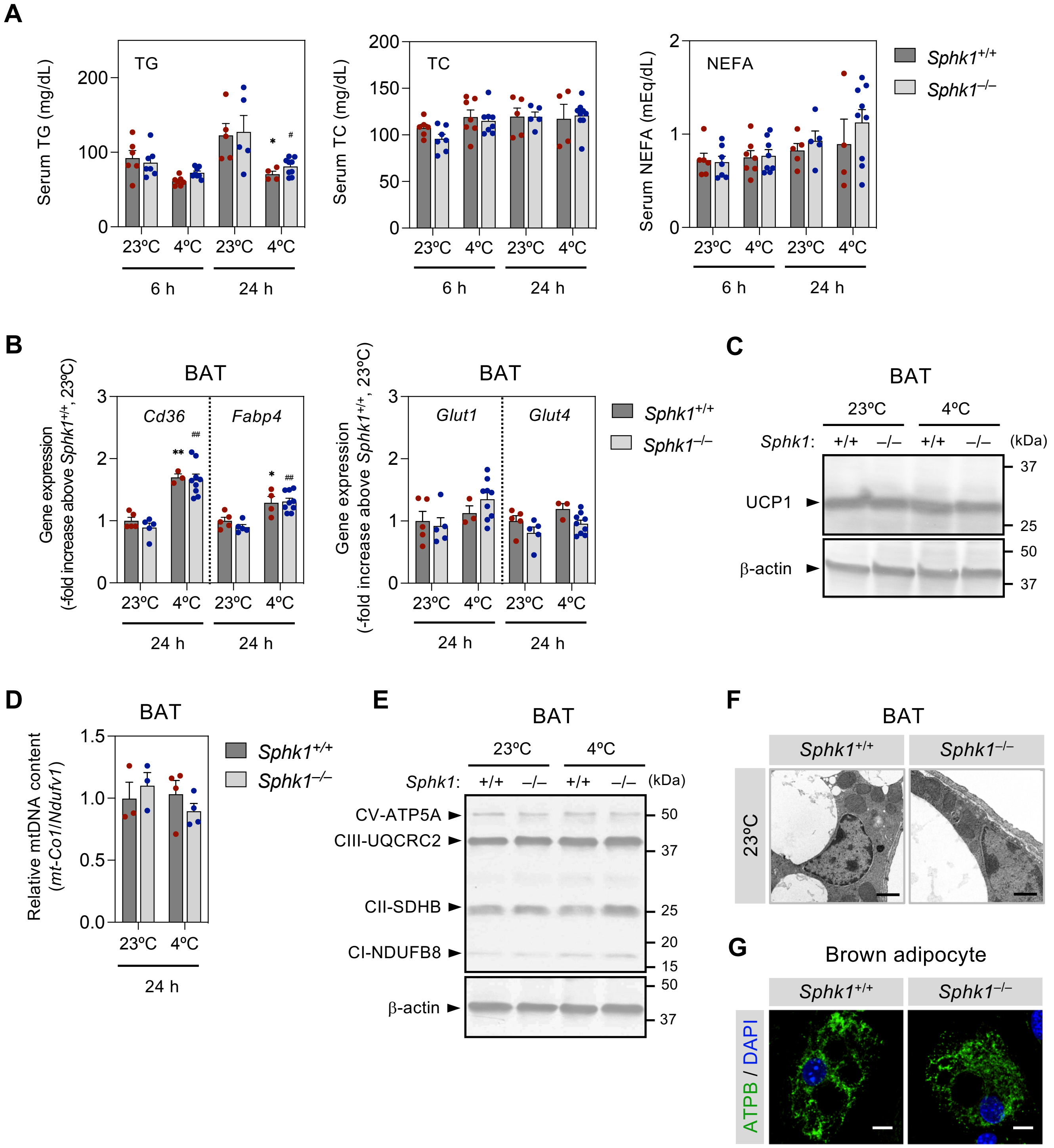

Figure S5. Comparable levels of fatty acid and glucose transporters and mitochondrial function in *Sphk1*$^{+/+}$ and *Sphk1*$^{-/-}$ BAT.

(A) Serum TG, total cholesterol (TC), and NEFA levels in *Sphk1*$^{+/+}$ and *Sphk1*$^{-/-}$ mice housed at 23°C or 4°C for 6 and 24 h (n = 4~9 mice per group). Data represent the mean ± SEM and analyzed by one-way ANOVA with Tukey's post hoc test. $^{*}P < 0.05$ versus *Sphk1*$^{+/+}$ (23°C, 24 h), $^{\#\#}P < 0.05$ versus *Sphk1*$^{-/-}$ (23°C, 24 h). (B) mRNA levels of fatty acid transporter (*Cd36* and *Fabp4*) and glucose transporter (*Glut1* and *Glut4*). mRNA levels are expressed relative to *Ppia* mRNA level in *Sphk1*$^{+/+}$ and *Sphk1*$^{-/-}$ mice housed at 23°C (n = 4~13 mice per group). Data represent the mean ± SEM and analyzed by two-way ANOVA with Tukey's post hoc test. $^{*}P <$ 0.05, $^{**}P < 0.01$ versus *Sphk1*$^{+/+}$ (23°C), $^{\#\#}P < 0.01$ versus *Sphk1*$^{-/-}$ (23°C). (C, D and E) Western blots of UCP1 (C), mitochondrial respiratory complexes protein (E), and relative mitochondrial DNA content (D) in BAT from *Sphk1*$^{+/+}$ and *Sphk1*$^{-/-}$ mice housed at 23°C or 4°C. As for loading control in (C), same β-actin image as in Figure 4A was used. Mitochondrial DNA (*mt-Co1*) contents are expressed relative to nuclear DNA (*Mdufv1*) (n = 3~4 mice per group). Data represent the mean ± SEM. (F and G) Transmission electron microscopy images of BAT from *Sphk1*$^{+/+}$ and *Sphk1*$^{-/-}$ mice housed at 23°C (F) and immunofluorescence images for ATPB in *Sphk1*$^{+/+}$ and *Sphk1*$^{-/-}$ brown adipocytes (G). Scale bar represents 2 μm and 10 μm, respectively.

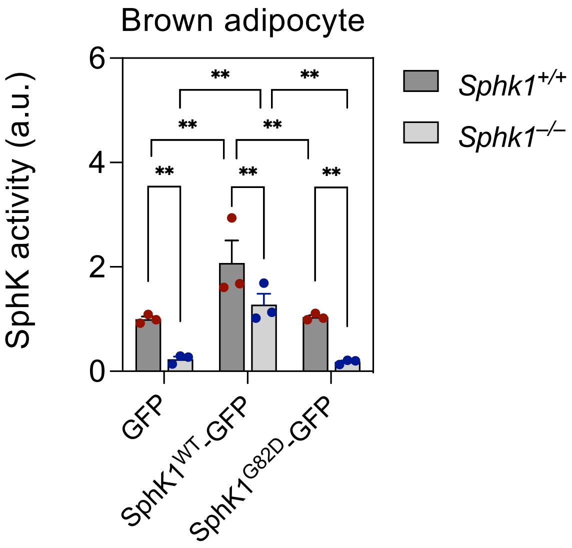

Figure S6. Effects of SphK1^{WT}-GFP and SphK1^{G82D}-GFP expression on SphK activity. SphK activity of cell lysate (20 µg of protein) prepared from *Sphk1*$^{+/+}$ and *Sphk1*$^{-/-}$ brown adipocytes transfected GFP, SphK1^{WT}-GFP, or SphK1^{G82D}-GFP vector (n = 3). Data represent the mean ± SEM and analyzed by two-way ANOVA with Tukey's post hoc test ($^{**}P < 0.01$).

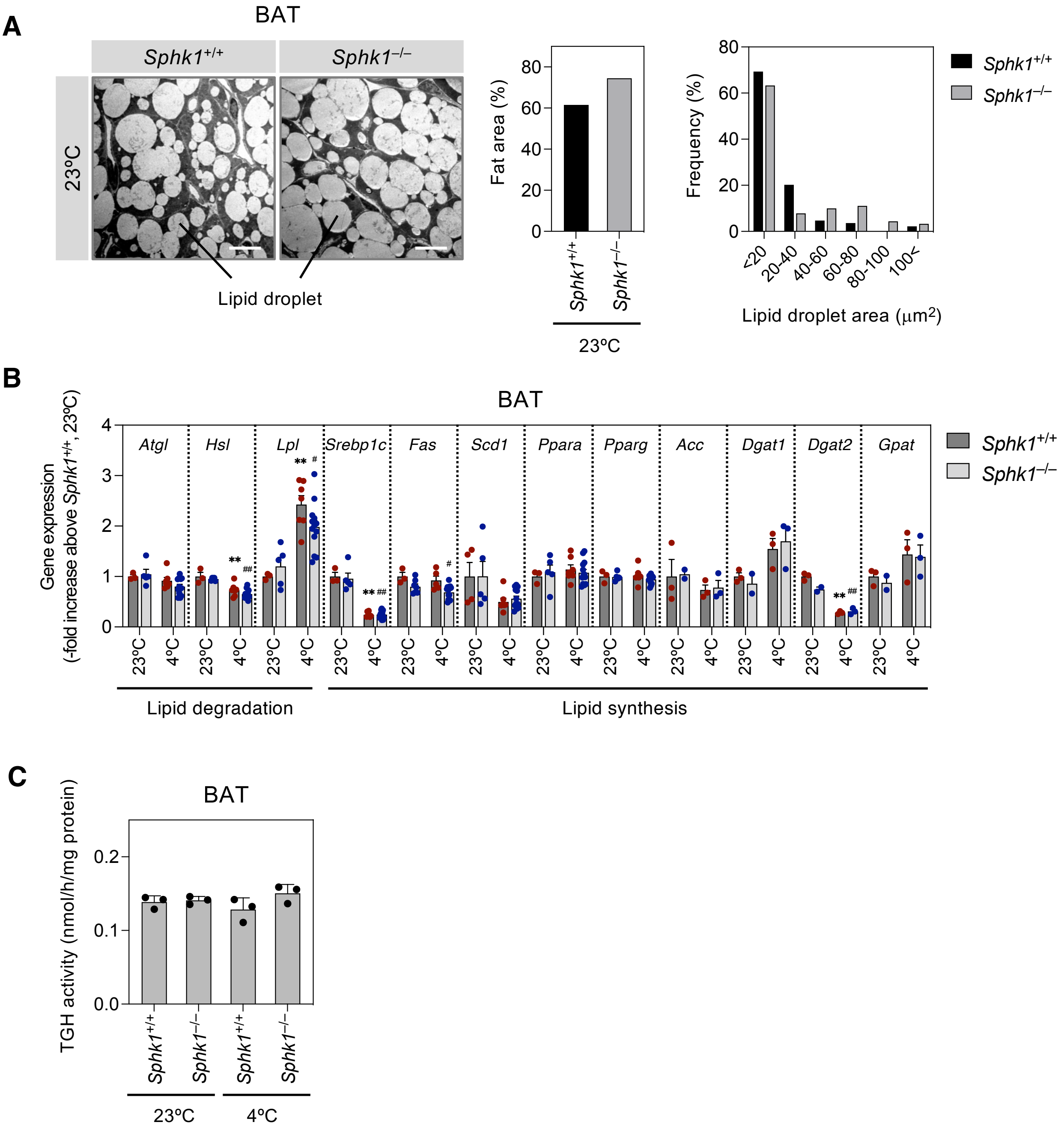

Figure S7. Lipid droplet size and mRNA levels of the enzymes involved in degradation and synthesis of TG in *Sphk1*$^{+/+}$ and *Sphk1*$^{-/-}$ BAT.

(A) Transmission electron microscopy images of BAT from *Sphk1*$^{+/+}$ and *Sphk1*$^{-/-}$ mice housed at 23ºC. Scale bar represents 10 µm. Histograms show the percentage fat area (left) and distribution of lipid droplet size (right) in the field. (B) mRNA levels of the enzymes engaged in degradation and synthesis of TG. mRNA levels are expressed relative to *Ppia* expression in *Sphk1*$^{+/+}$ mice housed at 23ºC (n = 4~13 mice per group). Data represent the mean ± SEM and analyzed by two-way ANOVA with Tukey's post hoc test. $^{**}P < 0.01$ versus *Sphk1*$^{+/+}$ (23ºC), $^{\#}P < 0.05$, $^{\#\#}P < 0.01$ versus *Sphk1*$^{-/-}$ (23ºC). (C) Neutral TG hydrolase activities of BAT lysate. Data represent the mean ± SEM. (n = 3).

Table S1. List of primer sequences used for qPCR

Gene	Forward (5' to 3')	Reverse (5' to 3')
Sphk1	AGGTGGTGAATGGGCTAATG	TGCTCGTACCCAGCATAGTG
Sphk2	ACTGCTCGCTTCTTCTCTGC	CACTGCACCCAGTGTGAATC
Sgpl1	TGCCATTCCTAAAGGTGGAC	CCCCATTGTACACAGCTCCT
S1p1r	AAATGCCCCAACGGAGACT	CTGATTTGCTGCGGCTAAATTC
S1p2r	GCCATCGTGGTGGAGAATCTT	AGGTACATTGCTGAGTGGAACTTG
S1p3r	GCGCATCTACTGCCTGGTCAAGTCC	AGCCAGCATGATGAACCACTGACTC
Ucp1	ACTGCCACACCTCCAGTCATT	CTTTGCCTCACTCAGGATTGG
Pgc1a	GAATCAAGCCACTACAGACACCG	CATCCCTCTTGAGCCTTTCGTG
Cd36	AATGGCACAGACGCAGCCT	GGTTGTCTGGATTCTGGA
Fabp4	AAGACAGCTCCTCCTCGAAGGTT	TGACCAAATCCCCATTTACGC
Glut1	CGTGGCCATCTTCTCTGTCG	AGGCCGCAGTACACACCGAT
Glut4	GTGACTGGAACACTGGTCCTA	CCAGCCACGTTGCATTGTAG
Lamp1	TAGTGCCCACATTCAGCATCTCCA	TTCCACAGACCCAAACCTGTCACT
Lipa	TGTTGCTTTTCACCATTGGGA	CGCATGATTATCTCGGTCACA
Tfeb	GCGAGAGCTAACAGATGCTGA	CCGGTCATTGATGTTGAACC
Atp6v1h	GTTGCTGCTCACGATGTTGGAG	TGTAGCGAACCTGCTGGTCTTC
Atg12	TGGCCTCGGAACAGTTGTTTA	GGGCAAAGGACTGATTCACAT
Ctsb	TTAGCGCTCTCACTTCCACTACC	TGCTTGCTACCTTCCTCTGGTTA
Atgl	CGCCTTGCTGAGAATCACCAT	AGTGAGTGGCTGGTGAAAGGT
Hsl	CTGCTGACCATCAACCGAC	CGATGGAGAGAGTCTGCA
Lpl	GTACCTGAAGACTCGCTCTC	AGGGTGAAGGGAATGTTCTC
Srebp1c	GGAGCCATGGATTGCACATT	GGCCCGGGAAGTCACTGT
Fasn	AGAGACGTGTCACTCCTGGACTT	GCTGCGGAAACTTCAGAAAAT
Scd1	CATCATTCTCATGGTCCTGCT	CCCAGTCGTACACGTCATTTT
Ppara	GAGGGTTGACGTCAGTCAGG	GGTCACCTACGAGTGGCATT
Pparg	GCCCTTTGGTGACTTTATGG	CAGCAGGTTGTCTTGGATGT
Acc	TGGAGAGCCCCACACACA	TGACAGACTGATCGCAGAGAAAG
Dgat1	GTGCACAAGTGGTGCATCAG	CAGTGGGATCTGAGCCATC
Dgat2	ACACCTTCTGCACAGACTGC	TGCGATCTCCTGCCACCTTT
Gpat	CATCCTCTTTTGCCACAACAT	ACAGAATGTCTTTGCGTCCA
Ppai	CAAGACTGAATGGCTGGATG	ATGGGGTAGGGACGCTCTCC
mt-Co1	TGCTAGCCGCAGGCATTAC	GGGTGCCCAAAGAATCAGAAC
Ndufv1	CTTCCCCACTGGCCTCAAG	CCAAAACCCAGTGATCCAGC
