# Supplementary material for: Sphingosine kinase 1 is involved in triglyceride breakdown by maintaining lysosomal integrity in brown adipocytes
Source: J Lipid Res. 2023 Sep 24;64(11):100450. doi: 10.1016/j.jlr.2023.100450 (PMC10630120; doi:10.1016/j.jlr.2023.100450)
Supplement: Table S1 [file mmc2.pdf]

Table S1. List of primer sequences used for qPCR

Gene	Forward (5' to 3')	Reverse (5' to 3')
Sphk1	AGGTGGTGAATGGGCTAATG	TGCTCGTACCCAGCATAGTG
Sphk2	ACTGCTCGCTTCTTCTCTGC	CACTGCACCCAGTGTGAATC
Sgpl1	TGCCATTCCTAAAGGTGGAC	CCCCATTGTACACAGCTCCT
S1p1r	AAATGCCCCAACGGAGACT	CTGATTTGCTGCGGCTAAATTC
S1p2r	GCCATCGTGGTGGAGAATCTT	AGGTACATTGCTGAGTGGAACTTG
S1p3r	GCGCATCTACTGCCTGGTCAAGTCC	AGCCAGCATGATGAACCACTGACTC
Ucp1	ACTGCCACACCTCCAGTCATT	CTTTGCCTCACTCAGGATTGG
Pgc1a	GAATCAAGCCACTACAGACACCG	CATCCCTCTTGAGCCTTTCGTG
Cd36	AATGGCACAGACGCAGCCT	GGTTGTCTGGATTCTGGA
Fabp4	AAGACAGCTCCTCCTCGAAGGTT	TGACCAAATCCCCATTTACGC
Glut1	CGTGGCCATCTTCTCTGTCG	AGGCCGCAGTACACACCGAT
Glut4	GTGACTGGAACACTGGTCCTA	CCAGCCACGTTGCATTGTAG
Lamp1	TAGTGCCCACATTCAGCATCTCCA	TTCCACAGACCCAAACCTGTCACT
Lipa	TGTTGCTTTTCACCATTGGGA	CGCATGATTATCTCGGTCACA
Tfeb	GCGAGAGCTAACAGATGCTGA	CCGGTCATTGATGTTGAACC
Atp6v1h	GTTGCTGCTCACGATGTTGGAG	TGTAGCGAACCTGCTGGTCTTC
Atg12	TGGCCTCGGAACAGTTGTTTA	GGGCAAAGGACTGATTCACAT
Ctsb	TTAGCGCTCTCACTTCCACTACC	TGCTTGCTACCTTCCTCTGGTTA
Atgl	CGCCTTGCTGAGAATCACCAT	AGTGAGTGGCTGGTGAAAGGT
Hsl	CTGCTGACCATCAACCGAC	CGATGGAGAGAGTCTGCA
Lpl	GTACCTGAAGACTCGCTCTC	AGGGTGAAGGGAATGTTCTC
Srebp1c	GGAGCCATGGATTGCACATT	GGCCCGGGAAGTCACTGT
Fasn	AGAGACGTGTCACTCCTGGACTT	GCTGCGGAAACTTCAGAAAAT
Scd1	CATCATTCTCATGGTCCTGCT	CCCAGTCGTACACGTCATTTT
Ppara	GAGGGTTGACGTCAGTCAGG	GGTCACCTACGAGTGGCATT
Pparg	GCCCTTTGGTGACTTTATGG	CAGCAGGTTGTCTTGGATGT
Acc	TGGAGAGCCCCACACACA	TGACAGACTGATCGCAGAGAAAG
Dgat1	GTGCACAAGTGGTGCATCAG	CAGTGGGATCTGAGCCATC
Dgat2	ACACCTTCTGCACAGACTGC	TGCGATCTCCTGCCACCTTT
Gpat	CATCCTCTTTTGCCACAACAT	ACAGAATGTCTTTGCGTCCA
Ppai	CAAGACTGAATGGCTGGATG	ATGGGGTAGGGACGCTCTCC
Mtco1	TGCTAGCCGCAGGCATTAC	GGGTGCCCAAAGAATCAGAAC
Ndufv1	CTTCCCCACTGGCCTCAAG	CCAAAACCCAGTGATCCAGC
